# Integrative genomic, virulence, and transcriptomic analysis of emergent *Streptococcus dysgalactiae* subspecies *equisimilis* (SDSE) *emm* type *stG62647* isolates causing human infections

**DOI:** 10.1128/mbio.02578-24

**Published:** 2024-10-17

**Authors:** Jesus M. Eraso, Randall J. Olsen, S. Wesley Long, Ryan Gadd, Sarrah Boukthir, Ahmad Faili, Samer Kayal, James M. Musser

**Affiliations:** 1 Laboratory for Molecular and Translational Human Infectious Diseases Research, Center for Infectious Diseases, Houston Methodist Research Institute, Houston, Texas, USA; 2 Department of Pathology and Genomic Medicine, Houston Methodist Hospital, Houston, Texas, USA; 3 Department of Pathology and Laboratory Medicine, Weill Medical College of Cornell University, New York, New York, USA; 4 Department of Microbiology and Immunology, Weill Medical College of Cornell University, New York, New York, USA; 5 CHU de Rennes, Service de Bacteriologie-Hygiène Hospitalière, Rennes, France; 6 INSERM, CIC 1414, Rennes, France; 7 Université Rennes 1, Faculté de Médecine, Rennes, France; 8 Université Rennes 1, Faculté de Pharmacie, Rennes, France; 9 OSS-Oncogenesis, Stress, and Signaling, INSERM 1242, Rennes, France; University of Illinois Chicago, Chicago, Illinois, USA

**Keywords:** *Streptococcus dysgalactiae*, genomics, pathogenesis, emerging clone

## Abstract

*Streptococcus dysgalactiae* subspecies *equisimilis* (SDSE) is a Gram-positive bacterial pathogen that infects humans and is closely related to group A streptococcus (GAS). Compared with GAS, far less is known about SDSE pathobiology. Increased rates of invasive SDSE infections have recently been reported in many countries. One SDSE *emm* type (*stG62647*) is known to cause severe diseases, including necrotizing soft-tissue infections, endocarditis, and osteoarticular infections. To increase our understanding of the molecular pathogenesis of *stG62647* SDSE isolates causing human infections, we sequenced to closure the genomes of 120 *stG62647* SDSE isolates. The genomes varied in size from 2.1 to 2.24 Mb pairs. The great majority of *stG62647* isolates had *IS1548* integrated into the *silB* gene, thereby inactivating it. Regions of difference, such as mobile genetic elements, were the largest source of genomic diversity. All 120 *stG62647* isolates were assayed for virulence using a well-established mouse model of necrotizing myositis. An unexpectedly wide range of virulence was identified (20% to 95%), as assessed by near-mortality data. To explore the molecular mechanisms underlying virulence differences, we analyzed RNAseq transcriptome profiles for 38 *stG62647* isolates (comprising the 19 least and most virulent) grown *in vitro*. Genetic polymorphisms were identified from whole-genome sequence data. Collectively, the results suggest that these SDSE isolates use multiple genetic pathways to alter virulence phenotype. The data also suggest that human genetics and underlying medical conditions contribute to disease severity. Our study integrates genomic, mouse virulence, and RNAseq data to advance our understanding of SDSE pathobiology and its molecular pathogenesis.

**IMPORTANCE:**

This study integrated genomic sequencing, mouse virulence assays, and bacterial transcriptomic analysis to advance our understanding of the molecular mechanisms contributing to *Streptococcus dysgalactiae* subsp. *equisimilis emm* type *stG62647* pathogenesis. We tested a large cohort of genetically closely related *stG62647* isolates for virulence using an established mouse model of necrotizing myositis and discovered a broad spectrum of virulence phenotypes, with near-mortality rates ranging from 20% to 95%. This variation was unexpected, given their close genetic proximity. Transcriptome analysis of *stG62647* isolates responsible for the lowest and highest near-mortality rates suggested that these isolates used multiple molecular pathways to alter their virulence. In addition, some genes encoding transcriptional regulators and putative virulence factors likely contribute to SDSE *emm* type *stG62647* pathogenesis. These data underscore the complexity of pathogen–host interactions in an emerging SDSE clonal group.

## INTRODUCTION

*Streptococcus dysgalactiae* subspecies *equisimilis* (SDSE) is a Gram-positive bacterial pathogen that infects humans and a variety of animals, including pigs, cows, sheep, horses, dogs, fish, and skunks (1–5). Whole genome sequencing has shown that SDSE isolates are composed of several host-specific sublineages, indicating that cross-species transmission is rare (6). The various host-specific sublineages have been reported to have distinct arrays of virulence factors, for example including adhesions and toxins that likely contribute to host specificity (6). SDSE isolates generally express Lancefield group C or G antigen, although some isolates can be group A or L. Genetic analysis has shown that SDSE is phylogenetic closely related to *Streptococcus pyogenes* (group A streptococcus; GAS) (7), likely sharing a common ancestor, although the timing is not clear (8). Compared with GAS*,* relatively little is known about the genomic, transcriptomic, and molecular pathogenesis mechanisms of SDSE (9–11). Unlike GAS, few experimentally confirmed virulence factors have been identified in SDSE (10–15), although several have been proposed based on homology to GAS genes (16–22).

Similar to GAS, SDSE can cause a spectrum of human infections, ranging from mild pharyngitis, tonsillitis, and skin infections, to severe invasive infections (23, 24), such as necrotizing myositis (24, 25), bacteremia (26), osteoarticular infections (OAI) (21, 27), and toxic shock syndrome (STSS) (25, 28, 29). Rarer infections include pneumonia, meningitis, and endocarditis (22, 30–32). Although the exact disease burden of SDSE remains unquantified, increased rates of invasive SDSE infections have recently been reported in several countries (22, 28, 33, 34). Concurrently, the emergence of SDSE isolates resistant to key antibiotics, including macrolides, fluoroquinolones, erythromycin, clindamycin, tetracycline, chloramphenicol, and beta-lactams, is an increasing public health concern (22, 25, 35–41).

The recently emerged SDSE *emm* type (*stG62647*) has been repeatedly associated with severe clinical manifestations, including STSS and necrotizing soft-tissue infections, in several countries (9, 18, 28, 42–47). We recently examined the virulence attributes of three SDSE isolates, including two *emm* type *stG62647* and one *emm* type *stC74a* isolate, in a mouse model of necrotizing myositis (9). The two *stG62647* isolates were more virulent than the *stC74a* isolate in this infection model. The *stG62647* isolates were multilocus sequence type clonal complex 20 (CC20), whereas the *stC74a* isolate was a genetically distinct CC17. This observation is consistent with recent epidemiological reports indicating an increase in both the frequency and severity of human infections caused by *stG62647* isolates. However, our initial analysis was limited to only a few isolates.

The goal of the current study was to expand our understanding of the pathobiology of an emerging SDSE lineage using an integrated approach involving: (i) complete genomic sequencing of all 120 *emm* type *stG62647* isolates identified in a comprehensive sample of 499 isolates cultured from humans and recovered between 2010 and 2018 in French Brittany (9); (ii) virulence assessment of the 120 *stG62647* isolates using a well-established mouse model of necrotizing myositis; and (iii) comparative transcriptome analysis of a subset of 38 *stG62647* isolates (35 CC20 and 3 CC17) grown *in vitro*. This subset included the 19 isolates causing the lowest and 19 isolates causing the highest near-mortality rates in the mouse necrotizing myositis virulence assay. The data from this integrative analysis address several knowledge deficits about an important emerging human bacterial pathogen and provide new data on the emergent *stG62647* clonal group causing severe human infections.

## MATERIALS AND METHODS

### Bacterial isolates and growth media

SDSE isolates were grown *in vitro* in Todd–Hewitt broth (Becton, Dickinson; BD) supplemented with 0.2% yeast extract (THY medium). To ensure consistency and minimize variations in the transcriptome due to differences in growth media composition, all media were prepared from a single master lot. Todd–Hewitt Broth (Research Products International, Mount Prospect, IL) (3 × 500 g; lot 7274–7951) and Yeast Extract (Life Technologies Corporation, Miami, FL) (100 g; lot 2397659) were combined and mixed. Aliquots were dispensed to make liquid media (32 g/L of water) as needed. In total, 6 L of broth was prepared by autoclaving 2 L in three 4 L flasks (KIMAX Kimble), as described (48). For solid media, SDSE isolates were grown on trypticase soy agar plates supplemented with 5% sheep red blood cells (Becton, Dickinson and Company). RNAseq experiments were performed in triplicate on SDSE isolates harvested at mid-exponential growth phase (OD_600_ = 1).

### Whole genome sequencing

The SDSE genomes were sequenced with methods described previously (9, 48–50). Briefly, isolates were grown at 37°C with 5% CO_2_ either on tryptic soy agar with 5% sheep blood (Becton, Dickinson, Franklin Lakes, NJ) or in Todd–Hewitt broth with 2% (wt/vol) yeast extract (THY; Difco Laboratories, Franklin Lakes, NJ). For Illumina short-read sequencing, chromosomal DNA was isolated with the RNAdvance viral kit (Beckman Coulter, Brea, CA) and a BioMek i7 instrument (Beckman Coulter). Libraries were prepared with the NexteraXT kit (Illumina, San Diego, CA) and sequenced with a NovaSeq instrument (Illumina) using a 2 × 250 bp protocol. The average depth of coverage for the *stG62647* isolates was 382 (range, 157–1639-fold coverage). For Oxford Nanopore Technologies long-read sequencing, chromosomal DNA was isolated with a DNeasy blood and tissue kit (Qiagen, Germantown, MD). Libraries were prepared with either a native barcoding kit or rapid barcoding kit (Oxford Nanopore Technologies, UK) and sequenced with a GridION instrument using version R10.4 or R9.4.1 flow cells (Oxford Nanopore Technologies), respectively.

Phylogeny among the isolates was inferred by neighbor joining based on core chromosomal SNPs. Dendroscope (51) was used for the graphical representation of phylogenetic trees.

### Genome assembly, closure, and annotation

The bioinformatics pipeline used to close genome assemblies and process RNAseq data is shown in Fig. S1. Genomes were assembled from the short- and long-read sequencing data. Initially, FASTQ files were preprocessed using Trimmomatic 0.39 to adapter and quality trim and filter the short-read Illumina reads, and Filtlong 0.2.1 to lengthen and quality filter the long-read data (1 kb minimum length and 90% retention threshold). Subsequently, Unicycler 0.5.0 was used for hybrid assembly of genomes with Spades 3.15.4 (52). Racon 1.5.0 (53) was used for assembly and polishing using the “normal” Unicycler setting (54). Genomes that did not close and circularize with this approach were assembled as described above using Unicycler with the “bold” setting. For genomes that did not assemble using either hybrid approach, a long-read-first approach was taken using Trycycler, with Minimap2 v2.17-r941, Miniasm v0.3-r179, Raven 1.8.1, and Minipolish, as described previously (9). Trycycler assemblies were polished with Polypolish and POLCA (55) and annotated with PROKKA (56) using SDSE isolate *emm* type *stG62647* MGCS36044 as the reference genome (9) (Fig. S1). Artemis (57) was used to browse and manually curate the annotated genomes. Multilocus sequence types were determined using MLST (https://github.com/tseemann/mlst) relative to the PubMLST database (https://pubmlst.org/), *emm* types were determined using EmmTyper (https://github.com/MDU-PHL/emmtyper) relative to the CDC *emm* type database (https://www2.cdc.gov/vaccines/biotech/strepblast.asp).

### Virulence assessment of *emm*-type *stG62647* isolates in a mouse model of necrotizing myositis

Mouse necrotizing myositis virulence studies were performed as described previously (48, 58–60). All 120 *stG62647* SDSE isolates identified in our comprehensive collection of 499 isolates from French Brittany were included (Table S1). MCGS36089, which is genetically representative of the *stG62647* isolates recovered in French Brittany and was previously characterized for mouse virulence (9), was used as the virulence reference isolate. Frozen stocks of each isolate were prepared by growth in Todd–Hewitt broth supplemented with 2% w/v yeast extract (Difco Laboratories) and quantified by counting colony-forming units (CFUs) recovered from cultures. For the virulence assays, immunocompetent 3-week-old female CD1 mice weighing 10 g (Envigo Laboratories) were randomly assigned to strain treatment groups. Each mouse was inoculated in the right lower hind limb with 1 × 10^8^ CFUs in 100 µL (*n* = 20 mice/strain; 2,380 mice total). This inoculation dose was selected based on previous experiments establishing that 1 × 10^8^ CFUs of the reference strain MCGS36089 resulted in 50% near-mortality (9). To validate the initial mouse near-mortality findings, 19 isolates representing the highest and lowest mortality groups were re-tested using the same parameters (*n* = 20 mice/strain; 380 mice total). This strategy identified isolates with significantly increased or decreased virulence. The mouse sample size was determined using a power calculation with the following variables: α = 0.05, power (1-ß) =0.8, difference in survival rates between groups = 0.4, and ratio of group size = 1. After inoculation, mice were monitored at least daily, and near-mortality was determined with internationally recognized criteria (61). Survival data were plotted as Kaplan–Meier curves or percent near-mortality graphs, and statistically significant differences were determined using either the log-rank test or Mann–Whitney test, respectively (Prism10, GraphPad Software).

### RNAseq library preparation and sequencing

For RNAseq analysis, 38 *stG62647* isolates exhibiting a low- or high-virulence phenotype in the mouse model of necrotizing myositis were selected (9). Specifically, 19 isolates with near-mortality rates ranging between 20% and 30% were classified as low-virulence isolates, and 19 isolates with near-mortality rates ranging between 70% and 95% were classified as high-virulence isolates. For RNAseq library generation, these 38 isolates were grown *in vitro* in THY broth and analyzed in triplicate at mid-exponential growth phase (OD_600_ = 1). Briefly, 2.2 mL from each culture was added to 4.4 mL RNAProtect (Qiagen, Germantown, MD), incubated for 20 min at RT, centrifuged for 15 min at 4,000 rpm, and the pellets were frozen at −80°C. RNA was extracted with an RNeasy kit (Qiagen) according to the manufacturer’s instructions. Ribosomal RNA (rRNA) was depleted using the NEBNext rRNA depletion kit (bacteria) (NEB, Beverly, MA), and the ribodepleted RNA was purified with Agencourt RNAClean XP paramagnetic beads (Beckman Coulter, Brea, CA). The quality of the total RNA and rRNA-depleted RNA was evaluated with RNA Nano and Pico chips, respectively, on an Agilent 2100 Bioanalyzer (Agilent Technologies, Santa Clara, CA). RNA integrity numbers for the 114 RNA samples (38 isolates with three biological replicates) ranged from 9.5 to 10, and rRNA contamination was negligible.

A total of 114 cDNA sequencing libraries were generated from the rRNA-depleted RNA using the NEBNext Ultra II Directional RNA Library Preparation Kit for Illumina (NEB), according to manufacturer’s instructions. SPRIselect paramagnetic beads (Beckman Coulter) were used for product purification in all subsequent steps. The cDNA libraries were bar-coded with 48 indexed reverse primers from the NEBNext multiplex oligonucleotides for Illumina (NEB). The quality of cDNA libraries was evaluated with DNA ScreenTape on a TapeStation 2200 (Agilent Technologies). The concentration of each sample was measured fluorometrically with Qubit dsDNA Broad Range and High Sensitivity kits (Thermo Fisher Scientific, Waltham, MA). All sequencing libraries had a cDNA concentration ≥100 nM.

Resultant RNAseq cDNA libraries were sequenced with an Illumina NextSeq550 instrument. Fastq files were demultiplexed, sequencing data quality was checked with FastQC (62), and the mean per-base sequence quality was ≥Q30 for all libraries. Adapter contamination and read quality filtering were performed with Trimmomatic (63). The RNAseq reads were mapped to the reference CC20 strain MGCS36044 genome (9), which contains a complement of regions of difference (RODs) representative of most isolates in this *stG62647* sample using EDGE-Pro. In some cases, RNAseq reads required individual mapping to their corresponding isolate genome due to variation in ROD content (Fig. S1). Library replicates produced on average 22 million reads (Fig. S2), corresponding to 100% genomic gene content coverage. To reduce noise and enhance statistical power genes expressed at low levels (that is, with normalized counts <20, and in ≥60% of the samples) were excluded from the expression analysis.

### Analysis of RNAseq data

Individual genome annotations generated with PROKKA (56) provided the necessary fasta, ptt, and rnt files for read mapping with EDGEpro (64), which in turn generated the raw reads for differential expression (DE) analysis. DE analysis was performed with DESeq2 (65) (Fig. S1) using RStudio. The analysis in RStudio was streamlined with pcaExplorer (66) and ideal (67). Genes were considered to be differentially expressed based on a greater than 1.5-fold change in transcript levels and an adjusted *P* value (*padj;* Benjamini–Hochberg corrected) of ≤0.05. rRNA and tRNA genes were excluded from the analysis. To compare CC17 and CC20 transcript levels, genes in RODs were also excluded due to the lack of shared RODs between CC17 isolates and the CC20 reference isolate MGCS36044.

### Identification of the core genome, accessory genome, and pangenome

The core, accessory, and pangenome gene content for the 38 *stG62647* isolates investigated was determined using Panaroo (68) (Fig. S1). SDSE putative virulence genes were inferred by BLAST search based on homology to a panel of known GAS virulence genes (Table S2).

### Polymorphism analysis

Illumina paired-end reads were mapped to the reference isolate MGCS36044 genome with SMALT (https://www.sanger.ac.uk/tool/smalt/), and polymorphisms between the aligned reads and the reference genome were identified with FreeBayes (https://doi.org/10.48550/arXiv.1207.3907). The statistical significance of the distribution of the polymorphisms between the low- and high-virulence isolate genomes was assessed with Pyseer and by χ analysis.

The lengths of homopolymeric T tracts in the genome of reference strain MGCS36044 were analyzed with the “find base pattern” function in Artemis (57), with an initial range of 20 consecutive T nucleotides, and decreasing to a final range of nine nucleotides (Table S3).

### Regions of difference and putative mobile genetic elements

Regions of variably present accessory gene content as defined by Panaroo were used to identify regions of difference (RODs) in consecutive gene content in all isolates. Subsequently, RODs unique to specific isolates or shared by multiple isolates were identified (Table 1). Sites of integration for putative MGEs, genes flanking the RODs identified with Artemis (57); genes flanking the RODs common to most of the isolates are shown in Table S4.

**TABLE 1:** Characteristics of the 38 *stG62647* isolates

Number	Isolate	% near- mortality	CC^*a*^	Virulence	Genome size in nt.	Number of locus tags	ROD count^*b*^	Number of genes in RODs	% ROD genes
1	MGCS35681	20	CC20	Low	2,171,406	2,221	15	249	11.2
2	MGCS35679	25	CC20	Low	2,105,313	2,150	13	191	8.9
3	MGCS35983	25	CC20	Low	2,156,048	2,211	14	244	11.0
4	MGCS35868	25	CC20	Low	2,156,576	2,206	14	246	11.2
5	MGCS35970	25	CC20	Low	2,157,490	2,220	14	243	10.9
6	MGCS35893	25	CC20	Low	2,215,594	2,292	15	315	13.7
7	MGCS35977	25	CC17^*c*^	Low	2,244,088	2,300	16	318	13.8
8	MGCS36023^*d*^	30	CC20	Low	2,156,090	2,206	14	246	11.2
9	MGCS35678	30	CC20	Low	2,156,317	2,203	14	244	11.1
10	MGCS35869	30	CC20	Low	2,158,434	2,208	14	243	11.0
11	MGCS35706	30	CC20	Low	2,160,476	2,217	14	244	11.0
12	MGCS35987	30	CC20	Low	2,167,341	2,227	15	252	11.3
13	MGCS35777	30	CC20	Low	2,172,722	2,223	15	249	11.2
14	MGCS36051	30	CC20	Low	2,202,749	2,246	16	276	12.3
15	MGCS36030	30	CC17	Low	2,203,793	2,230	15	242	10.9
16	MGCS35705	30	CC20	Low	2,203,864	2,297	17	319	13.9
17	MGCS35873	30	CC20	Low	2,205,608	2,258	15	294	13.0
18	MGCS36046	30	CC20	Low	2,219,796	2,311	16	331	14.3
19	MGCS36021^*e*^	30	CC17	Low	2,241,044	2,272	15	264	11.6
20	MGCS35941	70	CC20	High	2,155,982	2,202	14	246	11.2
21	MGCS35921	70	CC20	High	2,156,631	2,212	14	244	11.0
22	MGCS36067	70	CC20	High	2,156,854	2,207	14	245	11.1
23	MGCS35844	70	CC20	High	2,157,320	2,205	14	244	11.1
24	MGCS35838	70	CC20	High	2,157,501	2,214	14	244	11.0
25	MGCS35904	70	CC20	High	2,175,679	2,221	15	241	10.9
26	MGCS36035	70	CC20	High	2,199,191	2,276	15	314	13.8
27	MGCS36086	70	CC20	High	2,210,996	2,264	15	281	12.4
28	MGCS35730	75	CC20	High	2,109,976	2,169	13	194	8.9
29	MGCS36020	75	CC20	High	2,177,607	2,236	15	262	11.7
30	MGCS36061	75	CC20	High	2,195,165	2,239	15	282	12.6
31	MGCS36071^*f*^	80	CC20	High	2,156,154	2,206	14	247	11.2
32	MGCS35939	80	CC20	High	2,158,828	2,209	14	247	11.2
33	MGCS35957	80	CC20	High	2,233,554	2,326	17	327	14.1
34	MGCS35798	85	CC20	High	2,157,408	2,203	14	244	11.1
35	MGCS35978	85	CC20	High	2,159,390	2,214	14	245	11.1
36	MGCS35725	85	CC20	High	2,160,452	2,215	14	244	11.0
37	MGCS35785	90	CC20	High	2,157,613	2,207	14	244	11.1
38	MGCS35823	95	CC20	High	2,189,545	2,269	15	297	13.1
	**Min**.	**20**			**2,105,313**	**2,150**	**13**	**191**	**8.9**
	**Max**.	**95**			**2,244,088**	**2,326**	**17**	**331**	**14.3**

^*a*^ CC, clonal complex.

^*b*^ ROD, region of difference.

^*c*^ The CC17 isolates are separated from the CC20s by approximately 10,700 core SNPs.

^*d*^ MGCS36023 is one locus variant from CC20.

^*e*^ MGCS36021 is one locus variant from CC17.

^*f*^ MGCS36071 is one locus variant from CC20.

### Statistical analysis

The log-rank test was used to determine significant *P*-values for mouse survival data expressed as Kaplan–Meier curves and the Mann–Whiney test for percent near-mortality graphs (Table 1). Single-factor analysis of variance (ANOVA) was used for comparisons of mouse near-mortality (Fig. S3). DESeq2 was used to determine the adjusted *P*-value for differential expression analysis. Pyseer and χ were used to determine the significance of the difference in the distribution of polymorphisms (SNPs and InDels). χ analysis was used to assess the significance of differences in the distribution of the accessory gene content.

## RESULTS

### Genome sequence data

The study is based on a comprehensive analysis of all 499 SDSE isolates recovered from humans in French Brittany between 2010 and 2018. Illumina short-read sequencing found that *stG62647* was the most prevalent *emm* type (*n* = 120; 24%). These 120 isolates included organisms from invasive infections (*n* = 67), probably invasive infections (*n* = 11), non-invasive infections (*n* = 24), and carriers (*n* = 18). *Emm* type *stG62647* isolates have been associated with severe invasive infections and have recently increased in frequency across multiple countries (28, 42, 69, 70). Given their abundance in this French Brittany sample, their emergent nature, medical importance, and the relative lack of fundamental pathobiology data, we focused our study on the *stG62647* isolates.

**Fig 1:**
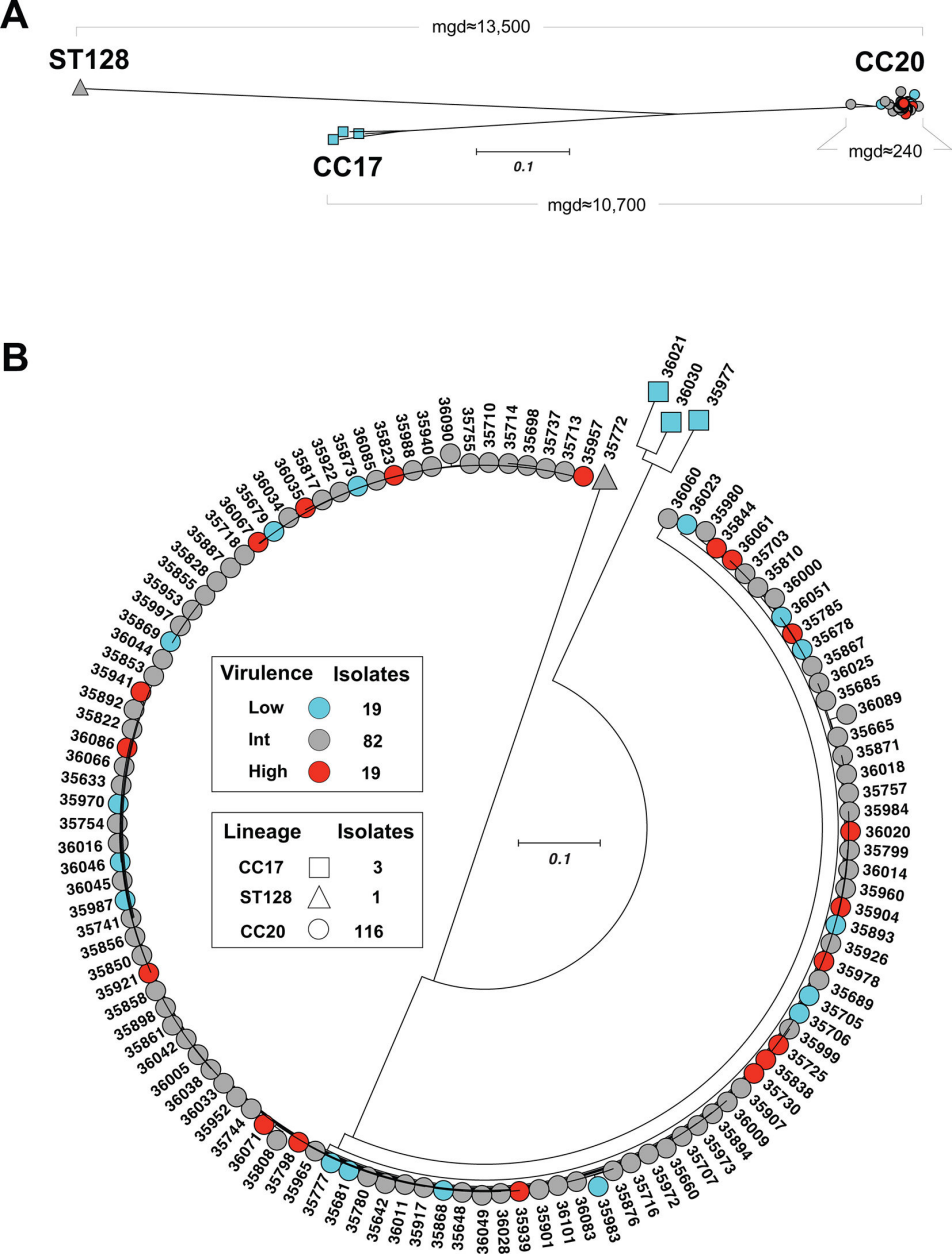
Genetic relationships among 120 SDSE *stG62647* isolates. The genetic relationships were inferred by neighbor joining based on 25,790 core SNPs defined relative to the reference isolate MGCS36044. (A) Three different genetic groups were identified. The 116 CC20 isolates clustered together and were separated, on average, by approximately 240 core-genome SNPs. The three CC17 isolates were separated, on average, by approximately 3,100 core-genome SNPs, and as a group were separated from the CC20s by approximately 10,700 core-genome SNPs. The ST128 isolate was separated from the CC20s by approximately 13,500 core-genome SNPs. mgd, mean genetic distance (in SNPs). (B) Radial phylogram depicting the 120 SDSE *stG62647* isolates. CC20 isolates ( = 116) are represented by circles, CC17s (*n* = 3) by squares, and the ST128 (*n* = 1) by a triangle. The virulence phenotype is shown in colors, as high virulence (red; *n* = 19), low virulence (blue; *n* = 19), and intermediate virulence (int, gray; *n* = 82).

To gain a fuller understanding of the population genomics of these *stG62647* isolates, we sequenced the genomes to closure by combining Illumina paired-end short-read and Oxford Nanopore (ONT) long-read data (Fig. S1). The vast majority of the *stG62647* isolates were multilocus sequence type clonal complex 20 (CC20; *n* = 116) and were closely related genetically, differing from each other by an average of only 240 core-genome SNPs (Fig. 1; Table S1). These data indicated that the great majority of French Brittany CC20 *stG62647* isolates are clonally related, descending from a relatively recent common ancestor. In contrast, three *stG62647* isolates were identified as clonal complex CC17, and one was *st128*, differing from the 116 CC20 isolates on average by 10,700 and 13,500 core-genome SNPs, respectively (Fig. 1).

### *stG62647* isolates differ widely in virulence in a mouse model of necrotizing myositis

**Fig 2:**
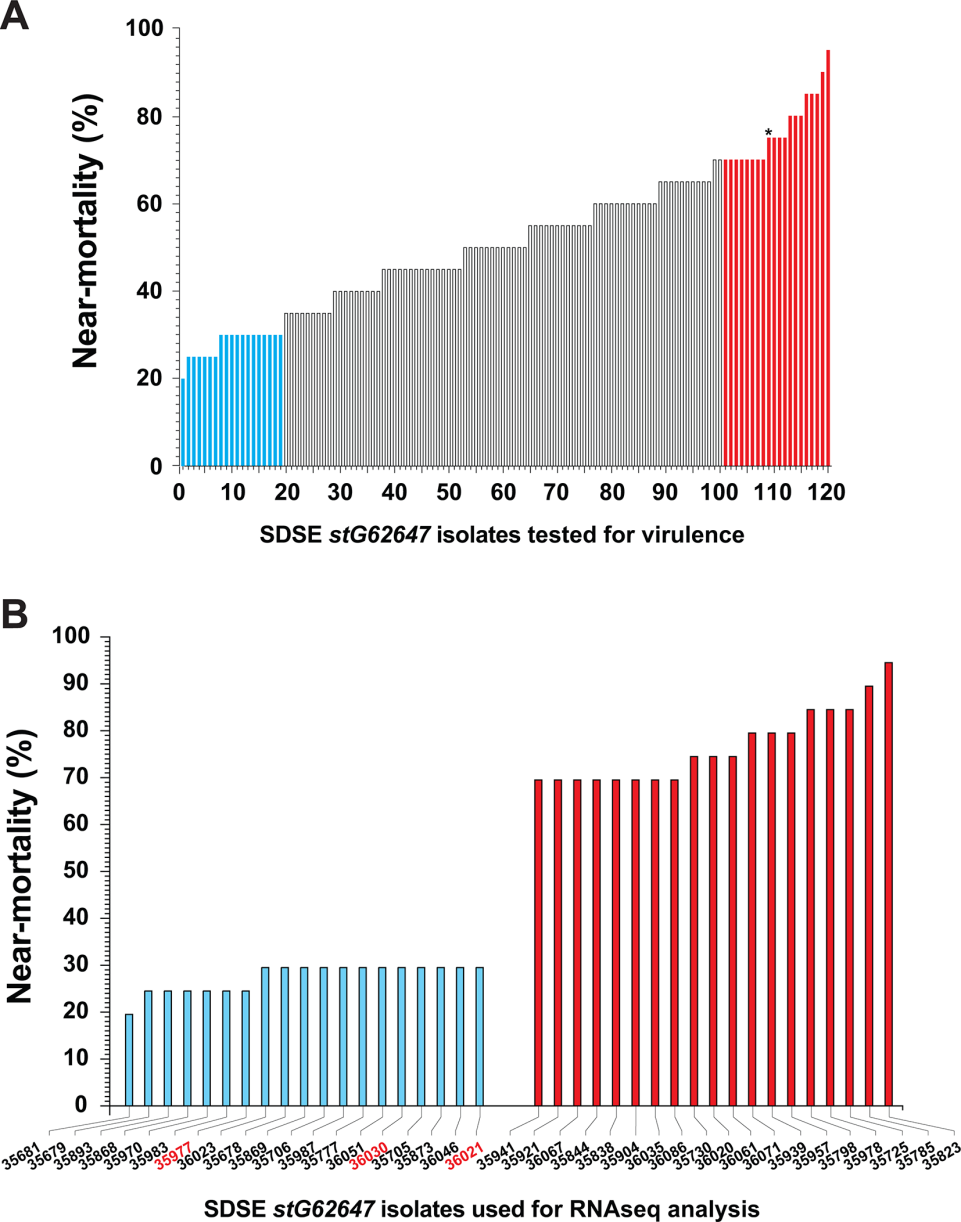
Mouse virulence of SDSE *stG62647* isolates. The 120 *emm*-type *stG62647* isolates from the comprehensive French Brittany collection of SDSE clinical isolates (*n* = 499) were inoculated into mice in the right lower hind limb (dose = 1×10^8^ CFUs; *n* = 20 mice/strain; 2,400 total mice). (A) Percent near-mortality for the 120 isolates. The isolates were separated into three virulence groups: (i) low virulence (blue bars; percent near-mortality [PM] = 20%–30%), (ii) isolates with intermediate virulence (white bars; PM = 35%–70%), and (iii) high-virulence strains (red bars; PM = 70%–95%). *, MGCS36044 was not assayed for RNAseq analysis. (B) Percent near-mortality values for isolates selected for RNAseq analysis. Thirty-five isolates were CC20 (black font), and three were CC17 (red font).

Despite most of the isolates differing modestly genetically, the 120 *emm*-type *stG62647* isolates had a wide range of virulence in a well-established mouse model of necrotizing myositis (48, 58–60) (Fig. 2; Table S1). Twenty mice each were inoculated in the lower hind limb with 1 × 10^8^ CFUs of each isolate. Mouse virulence varied considerably strain-to-strain, with near-mortality ranging from 20% to 95% (Fig. 2). As a group, the 19 isolates with highest near-mortality were significantly more virulent than the 19 isolates with lowest near-mortality (*P* < 0.0001, log-rank test). Among these 38 *stG62647* isolates, 35 were CC20, and three were CC17. Because of the unexpectedly wide range of near-mortality, we repeated the virulence assessment with 12 high-virulence isolates and seven low-virulence isolates, chosen from the 19 isolates in each corresponding group. The repeat near-mortality data confirmed the initial wide-range of mouse virulence.

The three CC17 *stG62647* isolates reproducibly had significantly lower mouse near-mortality compared with the 19 high-mortality CC20 isolates (Fig. S3).

### Analysis of *stG62647* closed-genome data

To identify potential molecular mechanisms underlying the substantial differences in mouse virulence, we analyzed the closed-genome data for the 38 *stG62647* isolates in the low- and high-virulence groups. Genomic and virulence characteristics for these 38 isolates are shown in Table 1. Their genomes varied in size from 2.16 to 2.24 Mbp and contained between 2,150 and 2,326 inferred genes. The variation in genome size and overall gene content was primarily due to discrete regions of difference (RODs), predominantly attributable to the integration of putative mobile genetic elements (MGEs). Among the 2,916 genes identified in the pangenome, 1,751 were present in all isolates and constitute the core genome, and 1,165 were variably present and constitute the accessory genome (Fig. S1). Importantly, in comparing the gene content of the low- and high-virulence isolates, no gene was found to be significantly non-randomly associated with either phenotypic group.

In total, 43 different RODs were identified across the 38 isolates, with each ROD containing between 4 and 51 inferred genes. Some RODs had gene content characteristic of integrative conjugative elements (ICEs), such as integrase/recombinase genes, or bacteriophage genes as described previously (9, 71). The core genes flanking the integrase genes in the RODs, including tRNA genes, *traG*, *rpmG*, and *rpsI*, were consistent with known streptococcal target genes for ICE insertion (71). Notably, several genes encoding small non-coding RNAs were also identified as putative ICE integration targets, an observation that may warrant future investigation (Table S4). In addition to putative ICE and bacteriophage genes, other ROD genes had the capacity to encode inferred transporters, transcriptional regulators, methyltransferases, toxin–antitoxin systems, and bacteriocins.

The number of RODs per isolate ranged from 13 to 17, comprising between 191 to 331 genes, accounting for 8.9% to 14.3% of each genome (Table 1). Isolates with the largest genomes had more RODs and corresponding accessory genes (Table 1). Consistent with the genomic differentiation between CC17 and CC20 isolates, only one ROD (36044 ROD.7 containing 50 genes on average) was shared between these two multilocus sequence-type groups. ROD.7 was present in one CC17 and two CC20 isolates. Twenty-five RODs were identified among the 35 CC20 isolates that were not present in the CC17 isolates. Fourteen had genes present in all 35 isolates, while the remaining were shared by only one to four isolates. Of the 18 RODs in the three CC17 isolates, 14 were present in all three isolates (Table S4). No individual gene was found significantly non-randomly distributed between the low- and high-virulence groups of isolates.

### RNAseq transcriptome analysis comparing low- and high-virulence SDSE isolates

To gain insight into how altered gene expression may contribute to differences in mouse virulence among *stG6264*7 isolates, we conducted RNAseq analysis on the 38 isolates with the lowest (*n* = 19) and highest (*n* = 19) mouse near-mortality groups. SDSE transcriptome data has only been done for a few isolates, largely focused on the two-component regulator CsrRS (aka CovRS). A pangenome-spotted microarray analysis was done for one strain and its *csrS* mutant derivative (11), and a genome-wide RNAseq analysis was done on one non-invasive human isolate and its *csrS* gene deletion mutant derivative (10). Importantly, no transcriptome studies have yet been conducted with SDSE *stG62647* isolates. Thus, this analysis provides much needed information on the transcriptome characteristics of these medically important emergent isolates.

The 38 SDSE isolates were grown in triplicate in THY broth and harvested at the mid-exponential growth phase. We used THY broth because (i) it has been previously used successfully in extensive transcriptomic studies of the genetically related pathogen GAS (48), and (ii) currently, there are no media that mimic *in vivo* growth in mammalian hosts.

**Fig 3:**
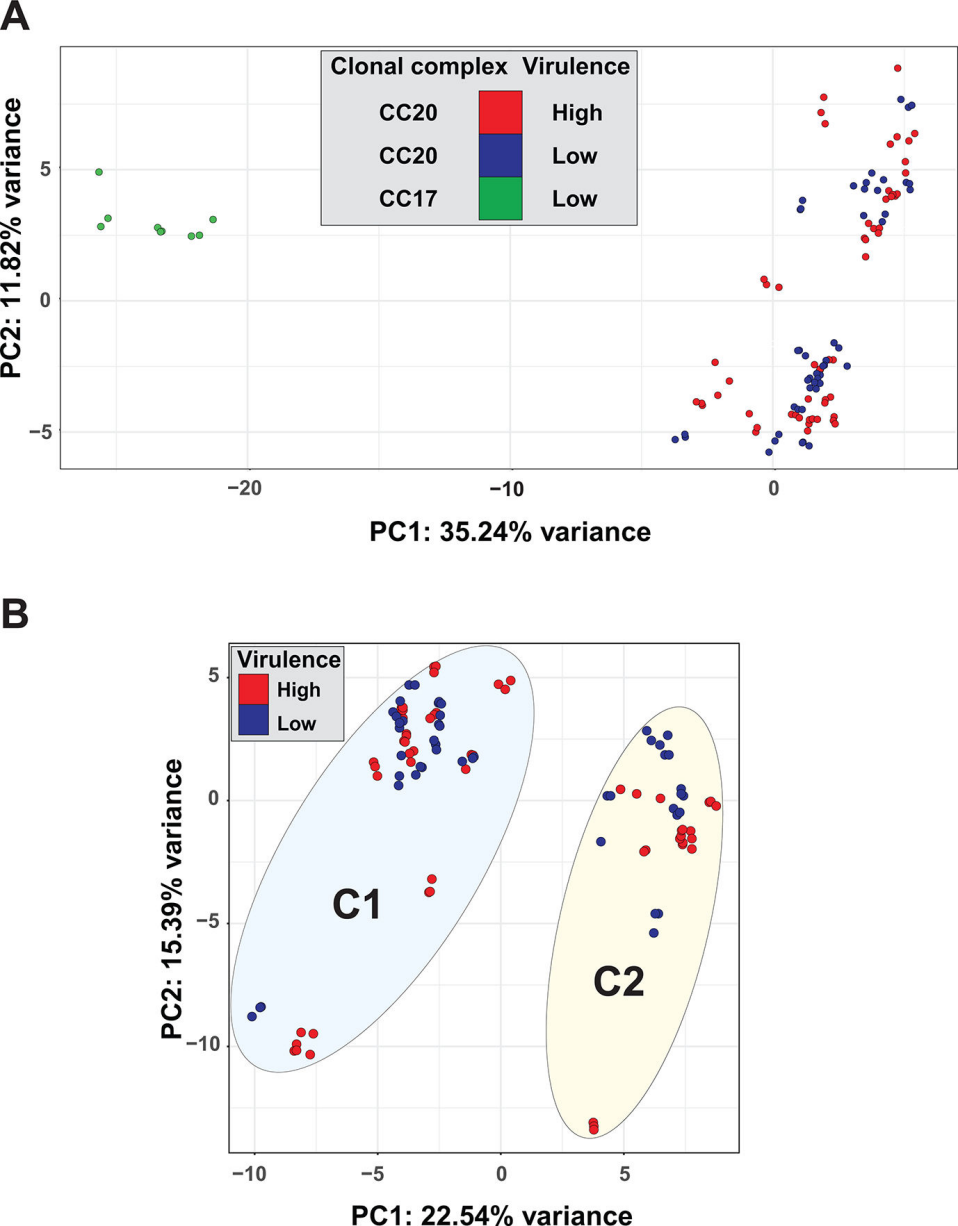
Transcriptome analysis of SDSE *stG62647* isolates grown *in vitro*: principal component analyses. SDSE *stG62647* isolates were grown *in vitro* in triplicate (114 replicates) to the mid-exponential phase. Thirty-five isolates were CC20, and three were CC17. (A) The CC17 replicates (green circles) clustered together and grouped distinctly apart from the CC20 replicates along principal component 1 (PC1). The CC20 high- (red circles) and low-virulence (blue circles) replicates were similarly interspersed along two somewhat distinct clusters along principal component 2 (PC2). (B) Upon exclusion of the three CC17 isolates, the CC20 replicates corresponding to 35 isolates were assigned to two distinct clusters designated C1 (*n* = 22 strains) and C2 (*n* = 13 strains), demarcated with ovals. The low- and high-virulence replicates were interspersed with each other.

To explore commonality in variation patterns in the RNAseq expression data set, principal component analysis (PCA) was conducted for these 38 isolates (38 isolates × 3 = 114 biological replicates) (Fig. 3A). First, for nearly all of the isolates, the three biological replicates were located in close proximity, indicating good within-isolate reproducibility. Second, the three CC17 isolates were well separated from the 35 CC20 isolates along principal component 1 (PC1). Among the 35 CC20 isolates two less distantly separated groups were found along PC2 (Fig. 3A). Importantly, these PCA parsings did not significantly correlate with virulence phenotype, as the CC20 low- and high-virulence isolates were interspersed with each other in the separate clusters.

Comparison of the transcriptome data from the CC17 and CC20 isolates identified 380 differentially expressed (DE) genes (at a fold-change [FC] ≥1.5 and an adjusted *P* ≤ 0.05), with 236 genes upregulated in the CC20 isolates, and 144 genes upregulated in the CC17 isolates. These DE genes included several putative virulence genes (Table S5). Despite the high reproducibility of the isolates in the RNAseq and mouse virulence data, the PCA clustering shows that there is no simple pattern of variance in gene expression shared by the *stG62647* isolates that correlate with differences in virulence phenotype.

### CC20 isolates separate into two distinct clusters by PCA

To further investigate why the CC20 isolates separate into two clusters differing in expression variance patterns (Fig. 3A), the analysis was repeated, omitting the three CC17 isolates. PCA parsed the 35 CC20 isolates (35 isolates × 3 = 105 biological replicates) into two well-resolved clusters along PC1 (Fig. 3B, C1 = 22 and C2 = 13). Again, when comparing the *stG62647* CC20 low- (*n* = 16) and high-virulence (*n* = 19) isolates as groups, the PCA clustering did not correlate with the virulence phenotype. Consistent with this lack of correlation, only 53 of 1,751 (3.0%) core genes were significantly DE between the virulence groups. Moreover, these 53 DE genes lacked any putative virulence factors. We interpret this lack of correlation to indicate that either (i) multiple different molecular pathways enable the *stG62647* CC20 isolates to acquire an altered virulence phenotype, or (ii) the *in vitro* growth conditions in THY broth do not adequately replicate the *in vivo* mouse necrotizing myositis growth environment. We note it is possible that *in vitro* growth to a different logarithmic phase may yield additional relevant data, but more extensive studies are required to address this idea.

**Fig 4:**
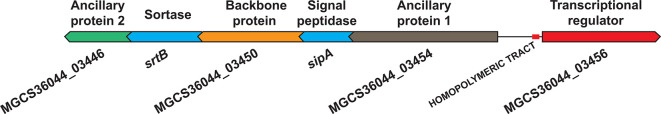
Organization of FCT-2 region genes. Genes and corresponding gene products in the FCT-2 region of the reference SDSE strain MGCS36044. From left to right: (i) MGCS36044_03446, pilus ancillary protein 2 (AP2); (ii) *srtB*, pilus sortase; (iii) MGCS36044_03450, pilus backbone protein (BP); (iv) *sipA*, signal peptidase; (v), MGCS36044_03454, pilus ancillary protein 1 (AP1); and (vi) MGCS36044_03456, putative transcriptional regulator. The location of the homopolymeric T-tract in the intergenic region is shown.

In contrast to the low number of DE genes identified when comparing the low- and high-virulence groups, when comparing the two *stG62647* CC20 PCA clusters, 409 genes were found significantly DE. Notably between C1 and C2, the most highly DE genes included five contiguous genes in the second of two fibronectin-collagen-T antigen (FCT-2) regions present in the SDSE genome (Fig. 4). These pilus-encoding genes had higher transcript levels in cluster 1 (C1 = high pilus expression) compared with cluster 2 (C2 = low pilus expression). This difference in expression was uniform, in that all five FCT-2 region genes were higher in expression in all 22 C1 isolates and lower in expression in all 13 C2 isolates. This observation, together with the molecular architecture of this region, suggests that these five contiguous genes comprise an operon (Fig. 4). An inferred stand-alone transcriptional regulator gene encoding an RofA-like protein (RALP) is divergently transcribed from these five genes analogous to pilus regulons in GAS (72, 73).

Analysis of the intergenic region between the divergently transcribed pilus genes and the RALP regulator gene identified a homopolymeric nucleotide tract (HPNT) of 14 Ts in the reference strain MGCS36044 genome. This HPNT is one of only two such HPNTs in the MGCS36044 genome that is longer than 10 Ts in length (Table S3), suggesting that this is rare and unlikely represents a random occurrence. The length of this HPNT varied from 9 to 19 Ts among the 35 CC20 *stG62647* isolates (Table 2). Importantly, all 13 CC20 C2 isolates with high transcript levels of the five DE pilus genes had an HPNT of either 13 or 14 Ts, whereas 21 of 22 CC20 C1 isolates with low transcript levels of the pilus genes had an HPNT either shorter or longer than 13–14 Ts in length. The exception was observed in isolate MGCS35970, where the putative RALP transcriptional regulator gene was disrupted due to a frame-shift mutation caused by the insertion of one T nucleotide in an intragenic HPNT (Table 2). This observation is consistent with this putative transcriptional regulator activating the expression of the five contiguous pilus genes.

**TABLE 2:** The length of the homopolymeric tract determines FCT-2 pilus gene expression

	Isolate	Length	Homopolymeric tract^*a*^	Virulence	Cluster^*b*^	Pilus gene expression^*c*^
1	MGCS35730	9	TTG**TTTTTTTTT**CTTTTACGA	HIigh	C1	Low
2	MGCS35844	9	TTG**TTTTTTTTT**CTTTTACGA	High	C1	Low
3	MGCS35904	9	TTG**TTTTTTTTT**CTTTTACGA	High	C1	Low
4	MGCS35798	11	TTG**TTTTTTTTTTT**CTTTTACGA	High	C1	Low
5	MGCS35838	11	TTG**TTTTTTTTTTT**CTTTTACGA	High	C1	Low
6	MGCS35777	12	TTG**TTTTTTTTTTTT**CTTTTACGA	Low	C1	Low
7	MGCS35939	12	TTG**TTTTTTTTTTTT**CTTTTACGA	High	C1	Low
8	MGCS35987	12	TTG**TTTTTTTTTTTT**CTTTTACGA	Low	C1	Low
9	MGCS36020	12	TTG**TTTTTTTTTTTT**CTTTTACGA	High	C1	Low
10	MGCS35970	13^*d*^	TTG**TTTTTTTTTTTTT**CTTTTACGA	Low	C1	Low
11	MGCS35681	15	TTG**TTTTTTTTTTTTTTT**CTTTTACGA	Low	C1	Low
12	MGCS35725	15	TTG**TTTTTTTTTTTTTTT**CTTTTACGA	High	C1	Low
13	MGCS35893	15	TTG**TTTTTTTTTTTTTTT**CTTTTACGA	Low	C1	Low
14	MGCS35957	15	TTG**TTTTTTTTTTTTTTT**CTTTTACGA	High	C1	Low
15	MGCS35978	15	TTG**TTTTTTTTTTTTTTT**CTTTTACGA	High	C1	Low
16	MGCS36023	15	TTG**TTTTTTTTTTTTTTT**CTTTTACGA	Low	C1	Low
17	MGCS36051	15	TTG**TTTTTTTTTTTTTTT**CTTTTACGA	Low	C1	Low
18	MGCS36071	15	TTG**TTTTTTTTTTTTTTT**CTTTTACGA	High	C1	Low
19	MGCS35706	16	TTG**TTTTTTTTTTTTTTTT**CTTTTACGA	Low	C1	Low
20	MGCS35983	16	TTG**TTTTTTTTTTTTTTTT**CTTTTACGA	Low	C1	Low
21	MGCS36086	16	TTG**TTTTTTTTTTTTTTTT**CTTTTACGA	High	C1	Low
22	MGCS35678	19	TTG**TTTTTTTTTTTTTTTTTTT**ACGA	Low	C1	Low
1	MGCS35705	13	TTG**TTTTTTTTTTTTT**CTTTTACGA	Low	C2	High
2	MGCS35873	13	TTG**TTTTTTTTTTTTT**CTTTTACGA	Low	C2	High
3	MGCS35921	13	TTG**TTTTTTTTTTTTT**CTTTTACGA	High	C2	High
4	MGCS35679	14	TTG**TTTTTTTTTTTTTT**CTTTTACGA	Low	C2	High
5	MGCS35785	14	TTG**TTTTTTTTTTTTTT**CTTTTACGA	High	C2	High
6	MGCS35823	14	TTG**TTTTTTTTTTTTTT**CTTTTACGA	High	C2	High
7	MGCS35868	14	TTG**TTTTTTTTTTTTTT**CTTTTACGA	Low	C2	High
8	MGCS35869	14	TTG**TTTTTTTTTTTTTT**CTTTTACGA	Low	C2	High
9	MGCS35941	14	TTG**TTTTTTTTTTTTTT**CTTTTACGA	High	C2	High
10	MGCS36035	14	TTG**TTTTTTTTTTTTTT**CTTTTACGA	High	C2	High
11	MGCS36046	14	TTG**TTTTTTTTTTTTTT**CTTTTACGA	Low	C2	High
12	MGCS36061	14	TTG**TTTTTTTTTTTTTT**CTTTTACGA	High	C2	High
13	MGCS36067	14	TTG**TTTTTTTTTTTTTT**CTTTTACGA	High	C2	High

^*a*^ Homololymeric tract in bold and flanking common nucleotides in all isolates.

^*b*^ Cluster refers to the two clusters of CC20 strains in the PCA plot in Fig. 3B.

^*c*^ Expression of the five consecutive pilus genes in the FCT-2 region.

^*d*^ Disrupted transcriptional regulator gene.

### RNAseq transcriptome analysis comparing individual strains within low- or high-virulence groups

To explore the hypothesis that the differences in virulence observed among the *stG62647* isolates might be multifactorial in nature, involving multiple different regulatory pathways, such that individual isolates achieve altered virulence following different routes, we reanalyzed the expression data using a one-against-all comparison approach. In this approach, we maintain an association with the virulence phenotype by assessing for gene content that an individual isolate of one virulence group differentially expresses relative to all isolates of the opposing virulence group. Therefore, we compared all 16 of the low-virulence CC20 isolates individually against all 19 high-virulence CC20 isolates as a group, and vice versa (*i.e*., 35 one-against-all comparisons).

**Fig 5:**
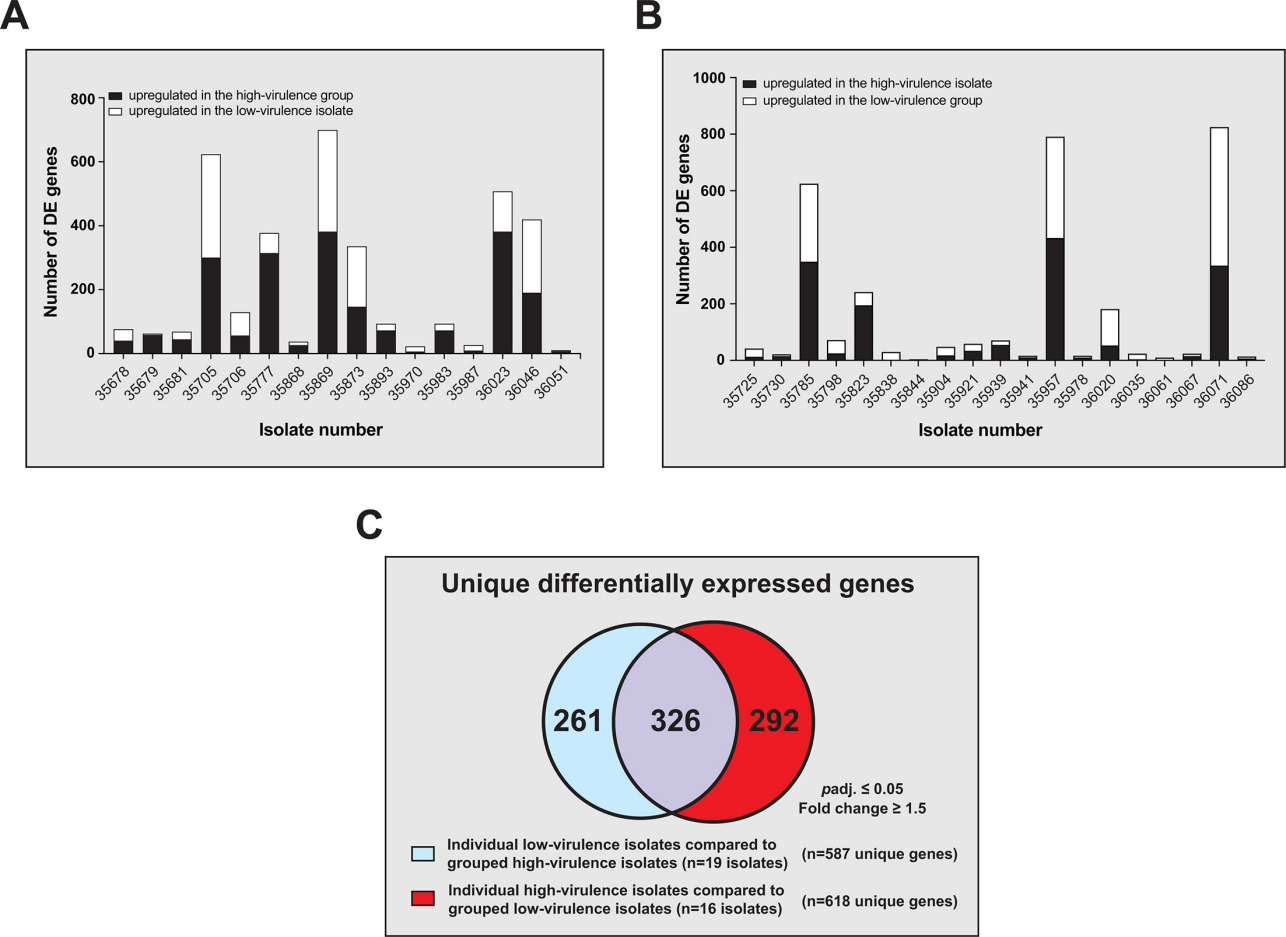
Summary of transcriptome analysis of SDSE *stG62647* CC20 isolates. SDSE *stG62647* isolates were grown in triplicate *in vitro* and collected at mid-exponential growth phase. The graphs represent the total number of differentially expressed genes in each comparison, either upregulated in the high-virulence isolates (black bars), or in the low-virulence isolates (white bars). The fold-change value cutoff and adjusted *P*-value cutoff was 1.5 and ≤0.05, respectively. (A) Sixteen comparisons for individual low-virulence isolates against grouped high-virulence isolates. (B) Nineteen comparisons for individual high-virulence isolates against grouped low-virulence isolates. (C) Venn diagram showing the number of unique differentially expressed genes, either for the individual low-virulence isolates compared with grouped high-virulence isolates (blue), or for the reciprocal set of comparisons (red). Genes present in both comparisons are shaded in purple.

Using a stringent cutoff for differential gene expression (*p*adj. ≤0.05, and FC ≥ 1.5-fold), the number of DE genes varied significantly among isolates (Fig. 5; Tables S6 and S7). For example, comparisons of individual low-virulence isolates to the grouped high-virulence isolates revealed DE genes ranging from 1 to 167, while the converse comparison yielded 1 to 216 DE genes. Importantly, regardless of the comparison, there were no common genes or biochemical pathways (as assessed by use of FUNAGE-Pro) (74) whose genes were differentially expressed across all comparisons. These data provide additional evidence for the existence of multiple molecular pathways for SDSE *stG62647* isolates to achieve a distinct mouse virulence phenotype. Possible mechanisms might include (i) polymorphisms in key genes, either in major transcriptional regulators (MTRs), such as those seen with *covS* in GAS (48, 75), or in genes encoding a virulence-related protein (60, 76), and/or (ii) through gene content differences in the accessory genome, which might not be apparent when sequencing reads from all isolates are mapped to a single reference isolate MGCS36044. Thus, we next investigated these possibilities.

### Polymorphism analysis

To test the hypothesis that nonsynonymous polymorphisms (nsSNPs) in MTR genes and putative virulence genes (Table S2) contribute to differences in mouse virulence among the SDSE *stG62647* isolates, we identified nsSNPs and Indels in the CC20 isolates (*n* = 35) relative to the genome of the CC20 reference isolate MGCS36044 (Table S8). Although nsSNPs were identified in genes known to regulate virulence in GAS (*e.g.*, *covRS* TCS, *mga*, and *fas* operon), nsSNPs in these genes were relatively uncommon, and there was no consistent association between nsSNPs or Indels and virulence phenotype in these isolates. Similarly, genome-wide analyses with Pyseer (77) also did not identify polymorphisms in genes significantly associated with differences in mouse virulence categories in this isolate sample.

### RNAseq transcriptome analysis of the RODs

We next tested the hypothesis that gene content differences in RODs contribute to mouse virulence phenotype. No ROD was uniquely present in either the low- or high-virulence groups and absent among isolates in the alternative virulence phenotypic category. The same was true for individual ROD genes. These results indicate that differences in ROD gene content between isolates are not responsible for the observed differences in mouse virulence.

We next analyzed differential gene expression in the RODs by comparing the 14 RODs shared by most CC20 isolates (encompassing a total of 244 genes), as two groups comprising low- or high-virulence isolates. The mean reads per kilobase of transcript per million (RPKM) values were calculated for all shared genes in a ROD, and fold-differences were calculated from the ratios of mean RPKM high virulence/mean RPKM low virulence. Nearly all of the 244 genes had mean RPKM ratios close to one when comparing the high- and low-virulence isolates. This lack of differential expression between the high- and low-virulence groups further reinforces that there are likely multiple molecular pathways responsible for altered virulence in this model.

## DISCUSSION

SDSE isolates of *emm*-type *stG62647* have recently emerged as a prominent cause of invasive human infections in multiple countries (9, 18, 28, 42–47). We discovered that *emm*-type *stG62647* isolates are the most common cause of SDSE infections in French Brittany, with a notable increase in occurrence over the 8-year study period (Fig. S4). These observations, coupled with the limited understanding of the molecular mechanisms contributing to SDSE pathogenesis, motivated this integrative study of the genomics, virulence, and transcriptomics of *stG62647* isolates.

We sequenced to closure the genomes of 120 *stG62647* isolates, including 116 CC20 and 3 CC17 organisms, thereby substantively expanding existing population genomics data for SDSE. These CC20 organisms are closely related, differing on average by only 240 core-genome SNPs (Fig. 1). The data indicate a relatively recent divergence from a common ancestor, although the exact timing is not known. Complete comparative genomic analysis of the 120 *stG62647* isolates was beyond the scope of this study but will be presented in a subsequent manuscript.

The genome sizes of the 38 SDSE isolates we analyzed were approximately 300–400 kbp larger on average than those of the closely related GAS genome. This increase is mainly due to the presence of discrete RODs in SDSE, some of which have gene content consistent with ICEs. The ROD content in these 38 *stG62647* isolates varied, but many RODs were shared among the majority of either CC20 or CC17 isolates.

In principle, these accessory genes could confer a survival advantage by expanding the coding capacity of the SDSE genomes. In this regard, we note that the 43 RODs included genes encoding inferred transporters, transcriptional regulators, methyltransferases, toxin–antitoxin systems, bacteriocins, and many hypothetical proteins. Additionally, as inferred from gene annotations, some of the inferred gene products have the potential to result in additional metabolic pathways or confer resistance to heavy metals, as previously suggested (78, 79).

Despite the close genomic relationships among the *stG62647* CC20 isolates in this SDSE sample, there was a strikingly wide range of virulence observed in a well-established mouse model of necrotizing myositis, a model that replicates many aspects of human disease caused by pathogenic beta-hemolytic streptococci (9, 48, 58, 59). We hypothesized the differences in virulence were caused, in part, by differences in gene expression, a phenomenon extensively documented in GAS (48, 60, 80). The *stG62647* transcriptome data revealed no simple relationship between mouse virulence and the overall pattern of *in vitro* gene expression. However, we did identify several potential molecular contributors to altered mouse virulence.

The 326 genes differentially expressed in both comparisons included several inferred putative virulence genes. Notable among these are genes important for SDSE virulence, such as streptolysin S region-encoding genes (10, 14), and *fasA*, which encodes the TCS response regulator FasA (14), albeit in different SDSE *emm* types. Additional differentially expressed putative virulence genes included the adhesin genes *fbp* and *fbpB*, encoding fibronectin-binding proteins; the transcriptional regulators *rofA* and *mga; emm*, which encodes the M protein; *slr,* which encodes an InlA-like protein; and *shr*, which encodes a heme-binding protein (Table S7). In addition, 38% of these common 326 differentially expressed genes are involved in carbohydrate and amino acid metabolism, consistent with a report that carbohydrate metabolism genes are differentially expressed during SDSE infection in an intraperitoneal mouse model (11).

Three additional SDSE genes or gene groups have been suggested or shown to affect SDSE virulence, including (i) disruption of *silB* in SDSE *stG62647* isolates (28); (ii) deletion of one of the three TTAAAGA heptanucleotide tandem direct repeats within *srrG* in SDSE *stG6792* isolates (10); and (iii) inactivation of *covRS* (*csrRS*), also in SDSE *stG6792* isolates (10, 11). These changes are either known or postulated to enhance SDSE virulence. The results show that a virulence phenotype can be influenced *via* mutational polymorphisms in SDSE, a phenomenon well-documented in GAS (48, 60, 75, 76).

Consistent with the hypothesis that multiple molecular pathways contribute to altered mouse virulence phenotype, our polymorphism analysis confirmed that there was no single polymorphism present in all isolates either in the low- or high-virulence group and absent in the alternative group. Given the numerous polymorphisms identified among the 35 CC20 isolates (CC17 isolates were more genetically distant), we concentrated on two groups of genes most likely to affect virulence: (i) genes encoding putative virulence factors, and (ii) genes encoding major transcriptional regulators (MTRs). Many of the gene products identified have not been directly studied in SDSE; however, their involvement in virulence has been inferred from similar studies conducted in GAS (10, 11, 14, 16, 18–22, 81). We chose this approach based on DNA sequence similarity between GAS and SDSE and the overlap in human disease manifestations observed between GAS and SDSE.

Importantly, compared with other MTR genes involved in virulence, *covS* (*n* = 6), *covR* (*n* = 2), *fas* (*n* = 5), and *mga* (*n* = 3), exhibited the largest number of polymorphisms in this isolate cohort, which is consistent with previous reports implicating CovRS, Fas, and Mga in GAS virulence (48, 75, 82, 83). Interestingly, in cases where nonsynonymous polymorphisms occurred in the same gene (e.g., *covR* or *fas*) in both low- and high-virulence isolates, the SNPs were located at different gene positions in each virulence group. Previous studies on different SDSE *emm* types have reported covRS polymorphisms and the effect of this TCS on virulence (10, 11, 14), but the SNPs we identified in *stG62647* differ from those reports.

When individual isolates were compared with an isolate (MGCS35679) lacking MTR or virulence gene polymorphisms, isolates harboring specific mutations, including CovS V52G, Mga E309K, and RofA L202F, showed differences in gene expression. A substantial number of the differentially expressed genes identified in these comparisons have been previously identified as members of the CovRS, Mga, and RofA regulons in GAS, respectively (83–85). We hypothesize that additional polymorphisms found in this SDSE cohort could similarly influence virulence phenotype, although confirmation through creation and analysis of isogenic strains is required to definitively determine these effects.

Genetic variation in the *sil* operon and *srrG* gene has also been implicated in virulence (10, 28). Oppegaard et al. (28) reported that 18 of 19 *stG62647* isolates causing invasive infections in Norway had a *silB* gene disrupted by insertion of *IS1548*. This was an important observation as *silB* encodes a sensory kinase that has been reported to increase virulence when inactivated (86, 87). The investigators speculated that inactivation of *silB* by insertion of *IS1548* contributed to enhanced virulence of *stG62647* CC20 isolates (28). The presence of this *IS1548* insertion at the same location in *silB* in the great majority of the French Brittany *stG62647* CC20 isolates (Table S1) is consistent with genome sequence data, indicating that the *stG62647* CC20 isolates had a relatively recent clonal origin. Conversely, the three *stG62647* CC17 isolates lacked the *sil* locus, analogous to data reported previously (28). The *sil* locus transcript abundance values were essentially identical in both virulence groups, indicating that there was not an association between expression level and differences in virulence phenotype in this animal model.

For the *srrG* gene, all 35 low- and high-virulence CC20 *stG62647* isolates had deletion of one of the three TTAAAGA tandem repeats, which Ikebe *et al*. reported enhances *sagA* gene expression and increases mouse virulence (10). This mutation might contribute to the increased virulence observed in CC20 *stG62647* compared with other SDSE *emm* types, although it does not fully account for the low- or high-virulence phenotypes observed here. Of the three CC17 stG62647 isolates, two contained three TTAAAGA tandem repeats, and one had only two tandem repeats. Taken together, these data are consistent with there being multiple pathways for SDSE *stG62647* isolates to modulate virulence phenotype.

Excluding the three CC17 *stG62647* isolates from the transcriptome analysis allowed the CC20 *stG62647* isolates to cluster into two discrete groups (Fig. 3B). However, there was not a simple association between variance in gene expression and mouse virulence phenotype. The variance was driven mainly by differences in the transcript levels of five genes in the pilus FCT-2 region (32- to 35-fold difference between the two groups). We found a strong association between the length of a homopolymeric tract of T residues in the presumed regulatory region, located between the divergently transcribed five-gene pilus operon (Table 2), and a gene encoding a RALP-like protein typically found in streptococcal FCT regions (88). Homopolymeric tracts are highly variable in length (48, 76), and in this cohort of 35 CC20 35 isolates, they ranged from 9 to 19T residues. With a single exception, lengths of 13 and 14T residues were associated with high transcript levels for the five pilus genes; any other length resulted in lower expression. In isolate MGCS35970, which had low transcript levels of the FCT-2 pilus genes despite having a 13T length, the gene encoding the divergently transcribed putative RALP-like transcriptional regulator had a single nucleotide (T) deletion at position 346, a polymorphism resulting in an out of frame mutation in the coding region of the gene. These data are consistent with the role of this putative RALP as an activator of FCT-2 pilus gene expression. The lack of a uniform association between pilus gene expression and enhanced virulence mirrors observations in GAS studies, where increased pilus gene expression results in decreased virulence in mouse models of invasive infection (89, 90). This suggests that in SDSE, as in GAS, pilus expression may enhance colonization and carriage but paradoxically decrease virulence in invasive infections. However, further investigation using isogenic mutant strains will be required to test this idea.

To summarize, we integrated full genome sequencing, mouse virulence, and *in vitro* transcriptome data from a cohort of 38 human SDSE isolates of the *stG62647 emm* type from French Brittany. Our comprehensive analysis leads us to conclude that multiple molecular pathways contribute to the diverse virulence phenotypes observed in this emerging group of organisms, which is now responsible for a significant number of infections across several geographic areas. In addition, the data suggest that human genetics and underlying medical conditions contribute to the range of disease severity. More work is required to further define specific molecular factors that contribute to host–pathogen interactions in SDSE organisms. Given that the isolates are from a relatively limited geographic region (Brittany, France), analysis of *stG62647* SDSE from more widely dispersed areas is warranted and might contribute information to the generalizability of our findings.

## Data Availability

Genome sequences have been submitted to the National Center for Biotechnology Information (NCBI) under Bioproject accession number PRJNA925803 (CP158994-CP159107).

## SUPPLEMENTAL MATERIAL

Fig. S1 mbio.02578-24-s0001.eps Flowchart depicting processes for analysis of SDSE stG62647 genomes, mouse virulence, and transcriptomes.

Fig. S2 mbio.02578-24-s0002.eps Genome sequencing reads obtained for 38 SDSE stG62647 isolates.

Fig. S3 mbio.02578-24-s0003.eps Comparison of mouse near-mortality for CC17 and CC20 isolates.

Fig. S4 mbio.02578-24-s0004.eps Temporal distribution of stG62647 isolates within the 499 French Brittany clinical isolates.

Supplemental Tables mbio.02578-24-s0005.docx Tables S1–S8.

ASM does not own the copyrights to Supplemental Material that may be linked to, or accessed through, an article. The authors have granted ASM a non-exclusive, world-wide license to publish the Supplemental Material files. Please contact the corresponding author directly for reuse.
