# Supplementary material for: Integrative genomic, virulence, and transcriptomic analysis of emergent Streptococcus dysgalactiae subspecies equisimilis (SDSE) emm type stG62647 isolates causing human infections
Source: mBio. 2024 Oct 17;15(11):e02578-24. doi: 10.1128/mbio.02578-24 (PMC11559094; doi:10.1128/mbio.02578-24)
Supplement: Supplemental Tables — Tables S1–S8. [file mbio.02578-24-s0005.docx]

**Table S1. 120 *stG62647* isolates tested for virulence**

| **No.** | **Isolate** | ***emm* type** | **% near-mortality** | **Virulence** | | **CC ^(1)^** | | ***IS1548***  **in *silB*** | **Sampling**  **site** | **Infection**  **type** |
| --- | --- | --- | --- | --- | --- | --- | --- | --- | --- | --- |
|  | 35681 | *stG62647* | 20 | low | CC20 | | Present | | Skin | noninvasive |
|  | 35679 | *stG62647* | 25 | low | CC20 | | Present | | Blood | invasive |
|  | 35893 | *stG62647* | 25 | low | CC20 | | Present | | Ano-genital | carriage |
|  | 35868 | *stG62647* | 25 | low | CC20 | | Present | | Blood | invasive |
|  | 35970 | *stG62647* | 25 | low | CC20 | | Present | | Pleuro-Pulmonary | noninvasive |
|  | 35983 | *stG62647* | 25 | low | CC20 | | Present | | Peritoneal | invasive |
|  | 35977 **^(2)^** | *stG62647* | 25 | low | CC17 | | Absent | | Bone | invasive |
|  | 36023 **^(3)^** | *stG62647* | 30 | low | CC20 | | Present | | Surgical sample | invasive |
|  | 35678 | *stG62647* | 30 | low | CC20 | | Present | | Blood | invasive |
|  | 35869 | *stG62647* | 30 | low | CC20 | | Present | | Skin/surgical | invasive |
|  | 35706 | *stG62647* | 30 | low | CC20 | | Present | | Pleuro-Pulmonary | probably  invasive |
|  | 35987 | *stG62647* | 30 | low | CC20 | | Present | | Blood | invasive |
|  | 35777 | *stG62647* | 30 | low | CC20 | | Present | | Skin | noninvasive |
|  | 36051 | *stG62647* | 30 | low | CC20 | | Present | | Urinary | carriage |
|  | 36030 **^(2)^** | *stG62647* | 30 | low | CC17 | | Absent | | Ano-genital | carriage |
|  | 35705 | *stG62647* | 30 | low | CC20 | | Present | | Bone | invasive |
|  | 35873 | *stG62647* | 30 | low | CC20 | | Present | | Skin | noninvasive |
|  | 36046 | *stG62647* | 30 | low | CC20 | | Present | | Blood | invasive |
|  | 36021 **^(2)^** | *stG62647* | 30 | low | CC17 | | Absent | | Skin/surgical | noninvasive |
|  | 35648 | *stG62647* | 35 | N/A **^(4)^** | CC20 | | Present | | Skin | probably  invasive |
|  | 35689 | *stG62647* | 35 | N/A | CC20 | | Present | | Bone | invasive |
|  | 35808 | *stG62647* | 35 | N/A | CC20 | | Present | | Skin | carriage |
|  | 35887 | *stG62647* | 35 | N/A | CC20 | | Present | | Bone | invasive |
|  | 35898 | *stG62647* | 35 | N/A | CC20 | | Present | | Blood | invasive |
|  | 35917 | *stG62647* | 35 | N/A | CC20 | | Present | | Ano-genital | carriage |
|  | 35965 | *stG62647* | 35 | N/A | CC20 | | Present | | Ano-genital | invasive |
|  | 35980 | *stG62647* | 35 | N/A | CC20 | | Present | | Synovial fluid | invasive |
|  | 36009 | *stG62647* | 35 | N/A | CC20 | | Present | | Bone | invasive |
|  | 35665 | *stG62647* | 40 | N/A | CC20 | | Present | | Blood | invasive |
|  | 35698 | *stG62647* | 40 | N/A | CC20 | | Present | | Blood | invasive |
|  | 35772 **^(5)^** | *stG62647* | 40 | N/A | ST128 | | Absent | | Synovial fluid | invasive |
|  | 35894 | *stG62647* | 40 | N/A | CC20 | | Present | | Blood | invasive |
|  | 35952 | *stG62647* | 40 | N/A | CC20 | | Present | | Blood | invasive |
|  | 35972 | *stG62647* | 40 | N/A | CC20 | | Present | | Pleuro-Pulmonary | invasive |
|  | 36005 | *stG62647* | 40 | N/A | CC20 | | Present | | Blood | invasive |
|  | 36011 | *stG62647* | 40 | N/A | CC20 | | Present | | Blood | invasive |
|  | 36060 | *stG62647* | 40 | N/A | CC20 | | Present | | Skin/surgical | noninvasive |
|  | 35642 | *stG62647* | 45 | N/A | CC20 | | Present | | Skin | noninvasive |
|  | 35707 | *stG62647* | 45 | N/A | CC20 | | Present | | Skin | noninvasive |
|  | 35817 | *stG62647* | 45 | N/A | CC20 | | Present | | Synovial fluid | invasive |
|  | 35822 | *stG62647* | 45 | N/A | CC20 | | Present | | Synovial fluid | invasive |
|  | 35850 | *stG62647* | 45 | N/A | CC20 | | Present | | ENT | noninvasive |
|  | 35855 | *stG62647* | 45 | N/A | CC20 | | Present | | Surgical sample | invasive |
|  | 35892 | *stG62647* | 45 | N/A | CC20 | | Present | | Urinary | carriage |
|  | 35901 | *stG62647* | 45 | N/A | CC20 | | Present | | Skin | noninvasive |
|  | 35960 | *stG62647* | 45 | N/A | CC20 | | Present | | Bone | carriage |
|  | 35988 | *stG62647* | 45 | N/A | CC20 | | Present | | Skin | invasive |
|  | 36018 | *stG62647* | 45 | N/A | CC20 | | Present | | Ano-genital | carriage |
|  | 36034 | *stG62647* | 45 | N/A | CC20 | | Present | | Synovial fluid | invasive |
|  | 36049 | *stG62647* | 45 | N/A | CC20 | | Present | | Ano-genital | probably  invasive |
|  | 36066 | *stG62647* | 45 | N/A | CC20 | | Present | | Skin/surgical | invasive |
|  | 36085 | *stG62647* | 45 | N/A | CC20 | | Present | | Urinary | carriage |
|  | 35633 | *stG62647* | 50 | N/A | CC20 | | Present | | Ano-genital | noninvasive |
|  | 35710 | *stG62647* | 50 | N/A | CC20 | | Present | | Skin | noninvasive |
|  | 35714 | *stG62647* | 50 | N/A | CC20 | | Present | | Bone | invasive |
|  | 35744 | *stG62647* | 50 | N/A | CC20 | | Present | | Blood | invasive |
|  | 35757 | *stG62647* | 50 | N/A | CC20 | | Present | | Oro-pharyngeal | probably  invasive |
|  | 35828 | *stG62647* | 50 | N/A | CC20 | | Present | | Synovial fluid | invasive |
|  | 35858 | *stG62647* | 50 | N/A | CC20 | | Present | | Blood | invasive |
|  | 35953 | *stG62647* | 50 | N/A | CC20 | | Present | | Skin | noninvasive |
|  | 35997 | *stG62647* | 50 | N/A | CC20 | | Present | | Peritoneal | invasive |
|  | 36014 | *stG62647* | 50 | N/A | CC20 | | Present | | Blood | invasive |
|  | 36025 | *stG62647* | 50 | N/A | CC20 | | Present | | Ano-genital | carriage |
|  | 36101 | *stG62647* | 50 | N/A | CC20 | | Present | | Skin | probably  invasive |
|  | 35660 | *stG62647* | 55 | N/A | CC20 | | Present | | Urinary | invasive |
|  | 35718 | *stG62647* | 55 | N/A | CC20 | | Present | | Skin | noninvasive |
|  | 35810 | *stG62647* | 55 | N/A | CC20 | | Present | | Blood | invasive |
|  | 35867 | *stG62647* | 55 | N/A | CC20 | | Present | | Skin/surgical | probably  invasive |
|  | 35871 | *stG62647* | 55 | N/A | CC20 | | Present | | Skin | probably  invasive |
|  | 35876 | *stG62647* | 55 | N/A | CC20 | | Present | | Ano-genital | carriage |
|  | 35907 | *stG62647* | 55 | N/A | CC20 | | Present | | Other | carriage |
|  | 35999 | *stG62647* | 55 | N/A | CC20 | | Present | | Oro-pharyngeal | carriage |
|  | 36000 | *stG62647* | 55 | N/A | CC20 | | Present | | Skin | noninvasive |
|  | 36042 | *stG62647* | 55 | N/A | CC20 | | Present | | Synovial fluid | invasive |
|  | 36045 | *stG62647* | 55 | N/A | CC20 | | Present | | Surgical sample | invasive |
|  | 36090 | *stG62647* | 55 | N/A | CC20 | | Present | | Blood | invasive |
|  | 36089 | *stG62647* | 60 | N/A | CC20 | | Present | | Bone | invasive |
|  | 35685 | *stG62647* | 60 | N/A | CC20 | | Present | | Urinary | invasive |
|  | 35703 | *stG62647* | 60 | N/A | CC20 | | Present | | Peritoneal | invasive |
|  | 35713 | *stG62647* | 60 | N/A | CC20 | | Present | | Skin/surgical | probably  invasive |
|  | 35737 | *stG62647* | 60 | N/A | CC20 | | Present | | Skin | invasive |
|  | 35754 | *stG62647* | 60 | N/A | CC20 | | Present | | Skin | noninvasive |
|  | 35755 | *stG62647* | 60 | N/A | CC20 | | Present | | Urinary | carriage |
|  | 35856 | *stG62647* | 60 | N/A | CC20 | | Present | | Skin | noninvasive |
|  | 35973 | *stG62647* | 60 | N/A | CC20 | | Present | | Blood | invasive |
|  | 35984 | *stG62647* | 60 | N/A | CC20 | | Present | | Other | carriage |
|  | 36028 | *stG62647* | 60 | N/A | CC20 | | Present | | Synovial fluid | invasive |
|  | 36083 | *stG62647* | 60 | N/A | CC20 | | Present | | Bone | invasive |
|  | 35716 | *stG62647* | 65 | N/A | CC20 | | Present | | Ano-genital | invasive |
|  | 35780 | *stG62647* | 65 | N/A | CC20 | | Present | | Bone | invasive |
|  | 35853 | *stG62647* | 65 | N/A | CC20 | | Present | | Blood | invasive |
|  | 35861 | *stG62647* | 65 | N/A | CC20 | | Present | | Synovial fluid | invasive |
|  | 35922 | *stG62647* | 65 | N/A | CC20 | | Present | | Urinary | carriage |
|  | 35926 | *stG62647* | 65 | N/A | CC20 | | Present | | Synovial fluid | invasive |
|  | 35940 **^(6)^** | *stG62647* | 65 | N/A | CC20 | | Absent | | Urinary | carriage |
|  | 36016 | *stG62647* | 65 | N/A | CC20 | | Present | | Blood | invasive |
|  | 36033 | *stG62647* | 65 | N/A | CC20 | | Present | | Blood | invasive |
|  | 36038 | *stG62647* | 65 | N/A | CC20 | | Present | | Ano-genital | noninvasive |
|  | 35741 | *stG62647* | 70 | N/A | CC20 | | Present | | Oro-pharyngeal | noninvasive |
|  | 35799 | *stG62647* | 70 | N/A | CC20 | | Present | | Oro-pharyngeal | probably  invasive |
|  | 35941 | *stG62647* | 70 | high | CC20 | | Present | | Synovial fluid | invasive |
|  | 35921 | *stG62647* | 70 | high | CC20 | | Present | | Blood | invasive |
|  | 36067 | *stG62647* | 70 | high | CC20 | | Present | | Bone | invasive |
|  | 35844 | *stG62647* | 70 | high | CC20 | | Present | | Skin | noninvasive |
|  | 35838 | *stG62647* | 70 | high | CC20 | | Present | | Skin | probably  invasive |
|  | 35904 | *stG62647* | 70 | high | CC20 | | Present | | Skin/surgical | noninvasive |
|  | 36035 | *stG62647* | 70 | high | CC20 | | Present | | Synovial fluid | invasive |
|  | 36086 | *stG62647* | 70 | high | CC20 | | Present | | Bone | invasive |
|  | 36044 **^(7)^** | *stG62647* | 75 | high | CC20 | | Present | | Bone | invasive |
|  | 35730 | *stG62647* | 75 | high | CC20 | | Present | | Skin | invasive |
|  | 36020 | *stG62647* | 75 | high | CC20 | | Present | | Skin | noninvasive |
|  | 36061 | *stG62647* | 75 | high | CC20 | | Present | | Urinary | invasive |
|  | 36071 **^(8)^** | *stG62647* | 80 | high | CC20 | | Present | | Ano-genital | noninvasive |
|  | 35939 | *stG62647* | 80 | high | CC20 | | Present | | Synovial fluid | invasive |
|  | 35957 | *stG62647* | 80 | high | CC20 | | Present | | Oro-pharyngeal | noninvasive |
|  | 35798 | *stG62647* | 85 | high | CC20 | | Present | | Blood | invasive |
|  | 35978 | *stG62647* | 85 | high | CC20 | | Present | | Synovial fluid | invasive |
|  | 35725 | *stG62647* | 85 | high | CC20 | | Present | | Pleuro-Pulmonary | probably  invasive |
|  | 35785 | *stG62647* | 90 | high | CC20 | | Present | | Urinary | carriage |
|  | 35823 | *stG62647* | 95 | high | CC20 | | Present | | Blood | invasive |

**^(1^**^)^ CC, clonal complex.

**^(2^**^)^ 35977, 36030 and 36021 do not contain a *sil* locus. 35977 and 36030 are CC17 and 36021 is one locus variant from CC17. The CC17 isolates are separated from the CC20s by ~10,700 core SNPs.

**^(3^**^)^ 36023 is one locus type variant from CC20.

**^(4^**^)^ N/A refers to not selected for transcriptome analysis.

**^(5^**^)^ 35772 is an ST128, and part of the *sil* locus, including *silA* and *silB*, is absent due to deletion. This isolate is ~13,500 core SNPs away from the CC20 isolates.

**^(6)^** The *IS1548* gene is absent but *silB* is 210-nt shorter at its 5’-end, compared to full-length *silB* from SDSE AC-2713, a Genbank strain with a wild-type *silB*.

**^(7)^** 36044 is the reference strain used for read mapping.

**^(8)^** 36071 is one locus type variant from CC20.

**Table S2. Putative genes associated with virulence in SDSE**

|  | **Gene_ID ^(1)^** | **Gene** | **Annotation** | | **References and comments** ^(2)^ |
| --- | --- | --- | --- | --- | --- |
| 1 | MGCS36044_01194 | ***ccpA*** | | catabolite control protein CcpA | [1] ^(^**^3^**^)^ |
| 2 | MGCS36044_02290 | ***ciaH*** | | TCS sensor histidine kinase protein CiaH | [1] |
| 3 | MGCS36044_02292 | ***ciaR*** | | TCS DNA-binding response regulator protein CiaR | [1] |
| 4 | MGCS36044_00878 | ***covR*** | | TCS DNA-binding response regulator CovR | [1-4] |
| 5 | MGCS36044_00880 | ***covS*** | | TCS sensor kinase CovS | [1-4] |
| 6 | MGCS36044_03700 | ***cppA*** | | CppA family putative C3-glycoprotein degrading | Homology to GAS virulence genes ^(^**^4^**^)^ |
| 7 | MGCS36044_00516 | ***emm*** | | cell surface M protein Emm | [1, 5, 6] |
| 8 | MGCS36044_03868 | ***fasA*** | | signal transduction response regulator protein | [1] |
| 9 | MGCS36044_03872 | ***fasB*** | | signal transduction sensor histidine kinase protein FasB | [1] |
| 10 | MGCS36044_03870 | ***fasC*** | | signal transduction sensor histidine kinase | [1] |
| 11 | MGCS36044_03866 | ***fasX*** | | FasABC signal tranduction system small | [1] |
| 12 | MGCS36044_00378 | ***fbp*** | | secreted fibronectin-binding protein | [5-9] |
| 13 | MGCS36044_03908 | ***fbpB* (*fbp_2*)** | | cell surface fibronectin binding protein (B) | Homology to GAS virulence genes |
| 14 | MGCS36044_03808 | ***gapA*** | | glyceraldehyde-3-phosphate dehydrogenase plasmin receptor adhesin GapA | [5, 7] |
| 15 | MGCS36044_00946 | ***hlyX*** | | hemolysin family protein HylX | Homology to GAS virulence genes |
| 16 | MGCS36044_04274 | ***htrA* (*degP*)** | | trypsin-like serine protease HtrA | [5, 7] |
| 17 | MGCS36044_01410 | ***hylB*** | | secreted hyaluronate lyase HylB | [5-8] |
| 18 | MGCS36044_02212 | ***hylIII*** | | membrane channel forming/hemolysin III protein | [6, 7] |
| 19 | MGCS36044_02050 | ***ihk*** | | TCS signal transduction histidine kinase sensor | [1] |
| 20 | MGCS36044_02048 | ***irr*** | | TCS signal transduction DNA-binding response | [1] |
| 21 | MGCS36044_03298 | ***liaF*** | | three component system signal transduction membrane component protein | Homology to GAS virulence genes |
| 22 | MGCS36044_03294 | ***liaR*** | | three component system signal transduction response regulator protein | Homology to GAS virulence genes |
| 23 | MGCS36044_03296 | ***liaS*** | | three component system signal transduction sensor histidine kinase protein | Homology to GAS virulence genes |
| 24 | MGCS36044_02062 | ***lmb*** | | laminin binding adhesin/bifunctional metal ABC transporter | [5-9] |
| 25 | MGCS36044_00514 | ***mga*** | | M protein trans-acting positive regulator Mga | [10] |
| 26 | MGCS36044_01062 | ***mtsR*** | | metal-dependent transcriptional regulator MtsR | [1] |
| 27 | MGCS36044_03998 | ***nga*** | | secreted nicotine adenine dinucleotide | [7] |
| 28 | MGCS36044_03944 | ***perR*** | | peroxide-responsive transcriptional repressor PerR | [1] |
| 29 | MGCS36044_00534 | ***pulA_1*** | | cell surface pullulanase PulA | [7] |
| 30 | MGCS36044_03456 | **RALPs** | | RofA-like protein type regulators | [1, 9] |
| 31 | MGCS36044_00362 | ***rofA*** | | pilus transcriptional regulator RofA | [1, 9] |
| 32 | MGCS36044_01510 | ***sagA*** | | streptolysin S precursor SagA | [2, 5, 6, 8] |
| 33 | MGCS36044_01514 | ***sagB*** | | streptolysin S biosynthesis protein SagB | [2, 6, 7] |
| 34 | MGCS36044_01516 | ***sagC*** | | streptolysin S biosynthesis protein SagC | [2, 6, 7] |
| 35 | MGCS36044_01518 | ***sagD*** | | streptolysin S biosynthesis protein SagD | [2, 6, 7] |
| 36 | MGCS36044_01520 | ***sagE*** | | streptolysin S self-immunity protein SagE | [2, 6, 7] |
| 37 | MGCS36044_01522 | ***sagF*** | | streptolysin S biosynthesis protein SagF | [2, 6, 7] |
| 38 | MGCS36044_01524 | ***sagG*** | | streptolysin S export protein SagG | [2, 6, 7] |
| 39 | MGCS36044_01526 | ***sagH*** | | streptolysin S export permease protein SagH | [2, 6, 7] |
| 40 | MGCS36044_01528 | ***sagI*** | | streptolysin S export permease protein SagI | [2, 6, 7] |
| 41 | MGCS36044_02058 | ***scpA_1*** | | cell surface extracellular C5a peptidase ScpA | [5-8] |
| 42 | MGCS36044_03614 | ***shr*** | | heme-, fibronectin-binding secreted protein Shr | [9] |
| 43 | MGCS36044_01126 | ***silA*** | | TCS DNA-binding response regulator SilA | [1] |
| 44 | MGCS36044_01132 | ***silB* ^(5)^** | | TCS histidine kinase SilB | [1] |
| 45 | MGCS36044_01134 | ***silCR*** | | streptococcal invasion locus auto-inducing pheromone peptide SilCR | [1] |
| 46 | MGCS36044_01138 | ***silD*** | | streptococcal invasion locus pheromone secretion accessory protein SilD | [1] |
| 47 | MGCS36044_01140 | ***silE*** | | streptococcal invasion locus pheromone processing peptide cleavage/export ABC transporter SilE | [1] |
| 48 | MGCS36044_00526 | ***ska*** | | secreted streptokinase Ska | [2, 5-8] |
| 49 | MGCS36044_03994 | ***slo*** | | secreted cholesterol-dependent cytolysin | [5-8] |
| 50 | MGCS36044_02866 | ***slr*** | | InlA-like streptococcal leucine rich lipoprotein | Homology to GAS virulence genes |
| 51 | MGCS36044_03926 | ***speG*** | | streptococcal pyrogenic exotoxin (G) SpeG | [7, 8] |
| 52 | MGCS36044_02840 | ***spg*** | | extracellular cell surface IgG-binding streptococcal protein (G) | [11] |
| 53 | MGCS36044_01508 | ***srrG*** | | streptolysin S small regulatory RNA SrrG | [3] |
| 54 | MGCS36044_03206 | ***trxR*** | | TCS DNA-binding response regulator protein TrxR | [1] |
| 55 | MGCS36044_03208 | ***trxS*** | | TCS sensor histidine kinase TrxS | [1] |
| 56 | MGCS36044_01238 | ***vicR*** | | TCS DNA-binding response regulator VicR | [1] |
| 57 | MGCS36044_01240 | ***vicK*** | | TCS signal transduction sensor kinase VicK | [1] |
| 58 | MGCS36044_03140 | ***yesM*** | | TCS sensor kinase YesM | [1] |
| 59 | MGCS36044_03142 | ***yesN*** | | TCS DNA-binding response regulator YesN | [1] |

**^(1)^** Locus tag designation for SDSE reference strain MGCS36044.

**^(2)^** Putative virulence genes in SDSE, referred to previously.

**^(3)^** Putative virulence genes in SDSE, based on homology to GAS genes.

**^(4)^** Putative virulence genes in SDSE, based on homology to GAS genes, by BLAST searches.

**^(5)^** *silB* is disrupted in MGCS36044.

| **Table S3. Homopolymeric T tracts in the MGCS36044 reference isolate** | | | | | | |  |
| --- | --- | --- | --- | --- | --- | --- | --- |
|  |  |  |  |  | |  | |
| **Number of Ts** | **number found** | **Genomic coordinates** | **Genomic location** | **Location with respect to a gene** | | **Function** | |
|  | **1** | 1,416,980 | core | upstream of *murA2* | | UDP-N-acetylglucosamine 1-carboxyvinyltransferase MurA | |
|  | 0 | N/A | core | N/A | | |  |
|  | 0 | N/A | core | N/A | |  | |
|  | 0 | N/A | core | N/A | |  | |
|  | **1** | 1,736,030 | core | upstream of 36044_03456 | | Transcriptional regulator in the FCT_2_ region **^(2)^** | |
|  | 0 | N/A | core | N/A | |  | |
|  | 0 | N/A | core | N/A | |  | |
|  | 0 | N/A | core | N/A | |  | |
|  | **1** | 1.511,314 | core | internal to *clpA* | | ATP-dependent Clp protease ATP-binding subunit ClpA | |
|  | 2 | 2,098,140 | ROD.9 **^(3)^** | upstream of 36044_04154 | | Hypothetical protein | |
|  | **1** | 184,371 | core | upstream of 36044_00434 | | Hypothetical protein | |
|  | 2 | 198,509 | ROD.2 | upstream of 36044_00470 | | Hypothetical protein | |
|  | 3 | 229,151 | core | downstream both from ska and 36044_00524 | | | |
|  | 4 | 916,979 | core | internal to *copB* | | Copper-exporting ATPase CopB | |
|  | 5 | 1,064,824 | core | internal to 36044_02180 | | PASTA domain-containing protein | |
|  | 6 | 1,097,701 | core | upstream of 36044_02246 | | Disrupted S8 family serine peptidase/extracellular C5a peptidase encoding gene | |
|  | 7 | 1,385,014 | ROD.7 | internal to 36044_02808 | | TetR/AcrR family transcriptional regulator | |
|  | 8 | 1,509,123 | core | upstream of *feoA* | | Ferrous iron transport protein (A) FeoA | |
|  | 9 | 1,579,654 | core | internal to *csd1* | | Csd1 family CRISPR-associated protein | |
|  | 10 | 1,866,472 | core | internal to 36044_03702 | | Putative sulfite exporter | |
| **^(1)^** Maximum number of T residues in a homopolymeric tract in the reference strain MGCS36044. A minimum number of 9 T residues was arbitrarily chosen as cutoff. | | | | | | | |
| **^(2)^** Highlighted in red because it is divergently transcribed from the pilus genes upregulated in G1 *stG62647* CC20 strains. | | | | | | | |
| **^(3)^** Designation for Regions Of Difference (ROD) in Beres *et al.* [12]. | | | | |  | | |

**Table S4. Genes flanking Regions of Difference present in most CC20 and CC17 clinical isolates**

| **CC ^(1)^** | **ROD ^(2)^** | | **# of genes ^(3)^** | **5' flanking**  **gene ^(4)^** | **Function/Annotation** | **3' flanking gene** | **Function/Annotation** | | **Integrase gene** |
| --- | --- | --- | --- | --- | --- | --- | --- | --- | --- |
| CC20 | RD.1 | 12 | | **tRNA-leu** | Leucine tRNA | **Spy392987** | *S. pyogenes* antisense RNA 392987 | Present | |
| CC20 | RD.2 | 10 | | ***-*** | PhoE family phosphoglycerate mutase | **tRNA-lys** | Lysine tRNA | Present | |
| CC20 | RD.3 | 42 | | ***rpsI*** | 30S ribosomal S9 protein RpsI | **-** | PRD domain/PTS transporter IIA domain protein | Present | |
| CC20 | RD.4 | 6 | | ***-*** | hypothetical protein | **-** | IS1182 family transposase | Absent | |
| CC20 | RD.5 | 7 | | ***-*** | IS3 family transposase ISLgar1 | **-** | BlpM-like bacteriocin with double-glycine leader peptide | Absent | |
| CC20 | RD.8 | 9 | | ***rgpB*** | glycosyltransferase family GT2 protein RgpB | ***galE*** | UDP-glucose 4-epimerase GalE | Absent | |
| CC20 | RD.10 | 53 | | ***rli38*** | regulator of virulence in Listeria RNA | ***dacA_3*** | secreted D,D-carboxypeptidase penicillin-binding protein DacA | Present | |
| CC20 | RD.11 | 18 | | ***-*** | IS1182 family transposase | **SpF66_sRNA** | *Streptococcus* sRNA SpF66 | Present | |
| CC20 | RD.12 | 5 | | ***yhhX*** | PRK10206 superfamily putative oxidoreductase | **-** | NusG domain II-containing protein | Present | |
| CC20 | RD.13 | 8 | | ***-*** | sigma-70 family RNA polymerase sigma factor like protein | ***topB*** | DNA topoisomerase III TopB | Absent | |
| CC20 | RD.14 | 21 | | ***traG_2*** | conjugal transfer protein TraG | ***-*** | LCP family anionic cell polymer synthesis enzyme | Present | |
| CC20 | RD.23 | 4 | | ***srtB*** | pilus polymerization class B sortase SrtB | ***-*** | IS982 family transposase | Absent | |
| CC20 | RD.24 | 27 | | ***-*** | Xre family helix-turn-helix transcriptional regulator | ***ybaB*** | YbaB family DNA-binding protein | Present | |
| CC20 | RD.25 | 19 | | ***rpmGA*** | 50S ribosomal L33 protein RpmGA | **-** | hypothetical protein | Present | |
| CC17 | RD.28 | 24 | | ***-*** | CYK3 family lipoprotein putatively invloved in cell division | **SRP** | small SRP RNA (4.5S RNA) | Absent | |
| CC17 | RD.29 | 13 | | ***ybaB*** | YbaB family DNA-binding protein | **Spy392987** | *S. pyogenes* antisense RNA 392987 | Present | |
| CC17 | RD.30 | 9 | | **-** | IS982 family transposase | **Spy392987** | *S. pyogenes* antisense RNA 392987 | Absent | |
| CC17 | RD.31 | 15 | | **-** | 23S rRNA (uracil(1939)-C(5))-methyltransferase RlmD | ***mobA*** | MobA/MobL family protein | Present | |
| CC17 | RD.32 | 18 | | ***topB_1*** | DNA topoisomerase III TopB | **-** | sigma-70 family RNA polymerase sigma factor like protein | Present | |
| CC17 | RD.33 | 9 | | **SpR18_sRNA** | Streptococcus sRNA SpR18 | **-** | IS1182 family transposase | Absent | |
| CC17 | RD.34 | 14 | | ***pemK*** | *pemK* motif RNA | ***rli38*** | regulator of virulence in Listeria RNA | Absent | |
| CC17 | RD.35 | 4 | | ***rimM*** | ribosome maturation factor RimM | **-** | KH domain-containing protein | Absent | |
| CC17 | RD.36 | 10 | | ***galE*** | UDP-glucose 4-epimerase GalE | ***rgpB*** | glycosyltransferase family GT2 protein RgpB | Absent | |
| CC17 | RD.37 | 31 | | ***tufA*** | translation elongation factor Tu protein TufA | **-** | sigma factor regulator | Absent | |
| CC17 | RD.38 | 4 | | **Spy392987** | *S. pyogenes* antisense RNA 392987 | **-** | hypothetical protein | Absent | |
| CC17 | RD.39 | 51 | | - | hypothetical protein | ***snf*** | SWF/SNF family helicase | Absent | |
| CC17 | RD.40 | 35 | | **-** | replication protein | ***rpsI*** | 30S ribosomal S9 protein RpsI | Present | |
| CC17 | RD.41 | 7 | | **-** | deoxyadenosine kinase | - | hypothetical protein | Absent | |

**^(1)^** CC, clonal complex.

**^(2)^** ROD, Region Of Difference. Only the RODs shared by most CC20 or CC17 isolates are shown. Amongst those that were not shared by most isolates, one was present in 4 isolates, two in three isolates, six in two isolates, and six in only one isolate.

**^(3)^** Genes refers to the number of genes present in each ROD.

**^(4)^** – refers to gene names not provided by the Prokka annotation.

**Table S5. Differentially expressed putative virulence genes comparing CC17 and CC20 isolates**

| **_(1)_** | **Locus tag** | **Gene ^(2)^** | | **FC ^(3)^** | **Pro/RNA ^(4)^** | **Annotation/Product** |
| --- | --- | --- | --- | --- | --- | --- |
| 1 | 36044_00378 | *fbpB* | 2.5 | | Pro | cell surface fibronectin binding protein (B) FbpB |
| 2 | 36044_00534 | *pulA_1* | -3.9 | | Pro | cell surface pullulanase PulA |
| 3 | 36044_01510 | *sagA* | 3.5 | | Pro | streptolysin S precursor SagA |
| 5 | 36044_01512 | *-* | 3.7 | | RNA | small RNA sagA |
| 6 | 36044_01514 | *sagB* | 4.0 | | Pro | streptolysin S biosynthesis protein SagB |
| 7 | 36044_01516 | *sagC* | 4.1 | | Pro | streptolysin S biosynthesis protein SagC |
| 8 | 36044_01518 | *sagD* | 4.1 | | Pro | streptolysin S biosynthesis protein SagD |
| 9 | 36044_01520 | *sagE* | 4.1 | | Pro | streptolysin S self-immunity protein SagE |
| 10 | 36044_01522 | *sagF* | 4.2 | | Pro | streptolysin S biosynthesis protein SagF |
| 11 | 36044_01524 | *sagG* | 4.1 | | Pro | streptolysin S export protein SagG |
| 12 | 36044_03926 | *speG* | 7.8 | | Pro | streptococcal pyrogenic exotoxin (G) SpeG |
| 13 | 36044_01526 | *sagH* | 4.2 | | Pro | streptolysin S export permease protein SagH |
| 14 | 36044_01528 | *sagI* | 4.0 | | Pro | streptolysin S export permease protein SagI |
| 15 | 36044_02062 | *lmb* | -2.3 | | Pro | bifunctional metal ABC transporter substrate-binding lipoprotein/laminin binding lipoprotein |
| 16 | 36044_03908 | *fbpB* | 6.8 | | Pro | cell surface fibronectin binding protein (B) FbpB |
| 17 | 36044_03926 | *speG* | 7.8 | | Pro | streptococcal pyrogenic exotoxin (G) SpeG |
|  |  |  |  | |  |  |

**^(1)^** Out of a total of 380 differentially expressed genes 17 encode putative virulence factors.

**^(2)^** Genes in the *sil* locus were excluded in this analysis since the CC17 isolates lack this genetic locus.

**^(3)^** FC refers to fold change calculated by DESeq2 as the ratio 35 CC20/3 CC17 isolates.

^(4)^ Pro and RNA refer to genes encoding proteins and RNA, respectively.

**Table S6. Differentially expressed genes in comparisons between individual isolates to grouped isolates from the opposite virulence phenotype**

| **No.** | **Virulence** | **Strain** | **DE genes by *padj.* ^(1)^** | **Up ^(2)^** | **Down** | **DE genes by *padj.* and fold ≥ 1.5** | **Up** | **Down** |
| --- | --- | --- | --- | --- | --- | --- | --- | --- |
| 1 | **Low ^(3)^** | **MGCS35678** | 76 | 40 | 36 | 18 | 0 | 18 |
| 2 | **Low** | **MGCS35679** | 62 | 58 | 4 | 35 | 34 | 1 |
| 3 | **Low** | **MGCS35681** | 68 | 44 | 24 | 15 | 10 | 5 |
| 4 | **Low** | **MGCS35705** | 623 | 300 | 323 | 137 | 13 | 124 |
| 5 | **Low** | **MGCS35706** | 129 | 56 | 73 | 34 | 3 | 31 |
| 6 | **Low** | **MGCS35777** | 377 | 314 | 63 | 84 | 59 | 25 |
| 7 | **Low** | **MGCS35868** | 37 | 26 | 11 | 3 | 1 | 2 |
| 8 | **Low** | **MGCS35869** | 699 | 381 | 318 | 167 | 84 | 83 |
| 9 | **Low** | **MGCS35873** | 335 | 146 | 189 | 111 | 21 | 90 |
| 10 | **Low** | **MGCS35893** | 93 | 72 | 21 | 45 | 41 | 4 |
| 11 | **Low** | **MGCS35970** | 21 | 6 | 16 | 12 | 0 | 12 |
| 12 | **Low** | **MGCS35983** | 93 | 72 | 21 | 45 | 41 | 4 |
| 13 | **Low** | **MGCS35987** | 26 | 9 | 17 | 9 | 0 | 9 |
| 14 | **Low** | **MGCS36023** | 507 | 381 | 126 | 154 | 53 | 101 |
| 15 | **Low** | **MGCS36046** | 419 | 190 | 229 | 105 | 51 | 54 |
| 16 | **Low** | **MGCS36051** | 10 | 7 | 3 | 1 | 1 | 0 |
| 1 | **High ^(4)^** | **MGCS35725** | 41 | 12 | 29 | 7 | 5 | 2 |
| 2 | **High** | **MGCS35730** | 21 | 14 | 7 | 17 | 14 | 3 |
| 3 | **High** | **MGCS35785** | 624 | 348 | 276 | 159 | 35 | 124 |
| 4 | **High** | **MGCS35798** | 71 | 24 | 47 | 50 | 8 | 42 |
| 5 | **High** | **MGCS35823** | 241 | 194 | 47 | 116 | 112 | 4 |
| 6 | **High** | **MGCS35838** | 29 | 1 | 28 | 13 | 0 | 13 |
| 7 | **High** | **MGCS35844** | 3 | 0 | 3 | 1 | 0 | 1 |
| 8 | **High** | **MGCS35904** | 47 | 17 | 30 | 21 | 0 | 21 |
| 9 | **High** | **MGCS35921** | 58 | 33 | 25 | 0 | 0 | 0 |
| 10 | **High** | **MGCS35939** | 70 | 54 | 16 | 11 | 8 | 3 |
| 11 | **High** | **MGCS35941** | 16 | 10 | 6 | 5 | 3 | 2 |
| 12 | **High** | **MGCS35957** | 790 | 432 | 358 | 216 | 96 | 120 |
| 13 | **High** | **MGCS35978** | 16 | 9 | 7 | 5 | 5 | 0 |
| 14 | **High** | **MGCS36020** | 181 | 52 | 129 | 48 | 27 | 21 |
| 15 | **High** | **MGCS36035** | 23 | 3 | 20 | 16 | 3 | 13 |
| 16 | **High** | **MGCS36061** | 9 | 1 | 8 | 4 | 1 | 3 |
| 17 | **High** | **MGCS36067** | 23 | 14 | 9 | 15 | 8 | 7 |
| 18 | **High** | **MGCS36071** | 824 | 334 | 490 | 214 | 97 | 117 |
| 19 | **High** | **MGCS36086** | 13 | 6 | 7 | 5 | 5 | 0 |

^(1)^ DE, differentially expressed. *padj.* ≤ 0.05.

^(2)^ The up and down terms are used with respect to the high-virulence isolates, either when analyzed individually or as a group.

**^(3)^** The comparison involved the corresponding low-virulence isolate *versus* 19 grouped high-virulence isolates.

**^(4)^** The comparison involved the corresponding high-virulence isolate *versus* 16 grouped low-virulence isolates.

**Table S7. Differentially expressed putative virulence genes in both sets of comparisons involving individual**

**isolates to grouped isolates from the opposite virulence phenotype**

| **No.** | **Locus tag ^(1)^** | **Gene** | **Annotation/Product** | **Frequency**  **^(2)^** | **Frequency ^(3)^** |
| --- | --- | --- | --- | --- | --- |
| 1 | 36044_00362 | ***rofA*** | **pilus transcriptional regulator RofA** | 2 | 3 |
| 2 | 36044_00378 | ***fbp*** | **secreted fibronectin-binding protein** | 2 | 3 |
| 3 | 36044_00514 | ***mga*** | **M protein trans-acting positive regulator Mga** | 1 | 3 |
| 4 | 36044_00516 | ***emm*** | **cell surface M protein Emm** | 1 | 3 |
| 5 | 36044_01510 | ***sagA*** | **streptolysin S precursor SagA** | 2 | 1 |
| 6 | 36044_01514 | ***sagB*** | **streptolysin S biosynthesis protein SagB** | 4 | 2 |
| 7 | 36044_01516 | ***sagC*** | **streptolysin S biosynthesis protein SagC** | 4 | 1 |
| 8 | 36044_01518 | ***sagD*** | **streptolysin S biosynthesis protein SagD** | 4 | 2 |
| 9 | 36044_01520 | ***sagE*** | **streptolysin S self-immunity protein SagE** | 4 | 2 |
| 10 | 36044_01522 | ***sagF*** | **streptolysin S biosynthesis protein SagF** | 2 | 2 |
| 11 | 36044_01524 | ***sagG*** | **streptolysin S export protein SagG** | 4 | 1 |
| 12 | 36044_01526 | ***sagH*** | **streptolysin S export permease protein SagH** | 4 | 1 |
| 13 | 36044_01528 | ***sagI*** | **streptolysin S export permease protein SagI** | 2 | 1 |
| 14 | 36044_02058 | ***scpA*** | **cell surface extracellular C5a peptidase ScpA** | 3 | 1 |
| 15 | 36044_02866 | ***slr*** | **InlA-like streptococcal leucine rich lipoprotein Slr** | 2 | 2 |
| 16 | 36044_03614 | ***shr*** | **heme-binding secreted protein Shr** | 1 | 1 |
| 17 | 36044_03868 | ***fasA*** | **signal transduction response regulator protein FasA** | 3 | 1 |
| 18 | 36044_03908 | ***fbpB*** | **cell surface fibronectin binding protein (B) FbpB** | 1 | 2 |

**^(1)^** Nomenclature for the reference strain MGCS36044

**^(2)^** Number of times the gene was differentially expressed in 16 individual low-virulence to grouped high-virulence isolates.

**^(3)^** Number of times the gene was differentially expressed in 19 individual high-virulence to grouped low-virulence isolates.

**Table S8. Amino acid replacements in putative virulence factors in 35 SDSE *stG62647* clonal**

**complex 20 isolates**

|  | **Isolate** | **Virulence** | **% near-**  **mortality** | **Amino acid replacements ^(1)^** |
| --- | --- | --- | --- | --- |
| 1 | **36086** | High | 70 | **ScpA** T1111A; **VicX** T41A; **Spg** E316Q; S365P; T368I |
| 2 | **36067** | High | 70 | - |
| 3 | **36035** | High | 70 | **Fbp** A1226V; **LiaS** G330R |
| 4 | **35941** | High | 70 | - |
| 5 | **35921** | High | 70 | **VicK** A440T; **Spg** T368I; S365P; E316Q |
| 6 | **35904** | High | 70 | **FasB** Y256C; **Fbp** D328A; **Irr** I112L |
| 7 | **35844** | High | 70 | - |
| 8 | **35838** | High | 70 | **RofA** L202F |
| 9 | **36061** | High | 75 | **Ihk** A29S; **Spg** Y274H |
| 10 | **36020** | High | 75 | **CovR** E125G; **Emm** E42K; **Slr** K585M |
| 11 | **35730** | High | 75 | **SagF** A93V; **CiaH** S215N |
| 12 | **36071** | High | 80 | - |
| 13 | **35957** | High | 80 | **CovS** V52G; **RofA** V127I; N305S |
| 14 | **35939** | High | 80 | **Mga** E432G; **Slr** S45T |
| 15 | **35978** | High | 85 | - |
| 16 | **35798** | High | 85 | - |
| 17 | **35725** | High | 85 | **CovS** I248M; E249A **Mga** A20T |
| 18 | **35785** | High | 90 | **Slr** H786Y |
| 19 | **35823** | High | 95 | **Mga** E309K |
|  |  |  |  |  |
| 1 | **35681** | Low | 20 | **YesM** V256G; **LiaF** K166E |
| 2 | **35679** | Low | 25 | - |
| 3 | **35868** | Low | 25 | **CovR** T73I; **Ska** E307G |
| 4 | **35893** | Low | 25 | ***fasB*** CT to C deletion at nt 794 **^(2)^** |
| 5 | **35970** | Low | 25 | **Spg** H53L |
| 6 | **35983** | Low | 25 | ***fasC*** C to CA insertion at nt 466 **^(2)^** |
| 7 | **35678** | Low | 30 | **FasA** I47V |
| 8 | **35705** | Low | 30 | - |
| 9 | **35706** | Low | 30 | **Mga** A20T; **CovS** I248M; E249A |
| 10 | **35777** | Low | 30 | **CovS** V196L; **YesM** V256G; **LiaF** K166E |
| 11 | **35869** | Low | 30 | **LiaR** L158R; **Slr** T195I; **ScpA** P1082T |
| 12 | **35873** | Low | 30 | **FasC** V271* **^(3)^** |
| 13 | **35987** | Low | 30 | **Spg** H53L; E316Q; S365P; T368I |
| 14 | **36023** | Low | 30 | **Emm** E42K |
| 15 | **36046** | Low | 30 | **CiaH** E319G; **Spg** H53L; E316Q; S365P; T368I |
| 16 | **36051** | Low | 30 | **Slr** H786Y |

**^(1)^** Only nonsynonymous substitutions are shown. Polymorphisms were called against the reference strain MGCS36044.

**^(2)^** The deletion and insertion in *fasB* and *fasC*, respectively, are indicated by the nucleotide position within the gene.

**^(3)^** A SNP resulted in an early STOP codon within *fasC*, generating a FasC truncation.
